# Targeting microglia-mediated neuroinflammation in Alzheimer’s disease: mechanisms and therapeutic approaches

**DOI:** 10.3389/fimmu.2026.1818802

**Published:** 2026-05-13

**Authors:** Bang-Feng Li, Xiao-Ying Chen, Rui Xie, Yi-Fei Mo, Yao-Zhu Wu, Ya Meng, Xiao-Ling Han, Mu-He Chen, Yong-Jun Peng

**Affiliations:** 1 Zhuhai People’s Hospital, The Affiliated Hospital of Beijing Institute of Technology, Zhuhai, Guangdong, China; 2 Guangdong Provincial Key Laboratory of Tumor Interventional Diagnosis and Treatment, Zhuhai Institute of Translational Medicine, Zhuhai, Guangdong, China; 3 Department of Medical Imaging, Zhuhai People’s Hospital (The Affiliated Hospital of Beijing Institute of Technology, Zhuhai Clinical Medical College of Jinan University), Zhuhai, Guangdong, China

**Keywords:** Alzheimer’s disease, immunotherapy, microglia, neurodegeneration, neuroinflammation

## Abstract

While the recent approval of amyloid-beta (Aβ)-clearing monoclonal antibodies (mAbs) marks a milestone in treating Alzheimer’s disease (AD), their modest clinical efficacy has catalyzed a paradigm shift, underscoring the necessity of targeting complementary pathological drivers. Neuroinflammation, once considered a secondary phenomenon, is now established as a third core pathological pillar of AD, with microglia at its epicenter. This review provides a comprehensive analysis of the multifaceted role of microglia in AD pathogenesis and evaluates the rapidly evolving landscape of microglia-targeted therapeutic strategies. We first delineate the dynamic and dichotomous function of microglia, which act as a “double-edged sword.” Emerging evidence reveals a complex, three-stage functional arc: microglia are implicated in the initial seeding of Aβ plaques, then transition to a neuroprotective role by containing established plaques, and finally devolve into a chronic, pro-inflammatory state that drives neurodegeneration. We then delve into the core molecular mechanisms governing this plasticity, including the pivotal Triggering Receptor Expressed on Myeloid Cells 2 (TREM2)-APOE signaling axis, the inhibitory receptor Cluster of Differentiation 33 (CD33), and key intracellular hubs like the NLRP3 inflammasome, which directly link genetic risk factors to microglial dysregulation. Based on this mechanistic understanding, we critically evaluate diverse therapeutic strategies, ranging from suppressing neurotoxic inflammation (e.g., TNF-α and NLRP3 inhibitors) to enhancing protective functions (e.g., TREM2 agonism and CD33 antagonism), eliminating senescent microglia (senolytics), and utilizing advanced nanoplatforms for brain-targeted delivery. Finally, we highlight the critical role of neuroinflammatory biomarkers within the emerging ATI(N) framework for enabling precision medicine. In conclusion, targeting microglia represents a vital therapeutic avenue that moves beyond amyloid-centric approaches, where a sophisticated understanding of their stage-dependent functions is paramount for developing effective immunomodulatory therapies to alter the devastating course of AD.

## 1 Introduction

For decades, the conceptual framework for Alzheimer’s disease (AD) has been dominated by the amyloid cascade hypothesis, which posits that the accumulation of amyloid-beta (Aβ) peptides is the primary pathological trigger, leading sequentially to tau pathology, synaptic dysfunction, and neurodegeneration (1, 2). This hypothesis has driven the development of numerous therapeutic agents aimed at reducing the Aβ burden. The recent approvals of anti-Aβ monoclonal antibodies (mAbs), such as aducanumab, lecanemab, and donanemab, represent a landmark achievement, as these therapies have demonstrated an unprecedented ability to clear amyloid plaques from the brain (3–5). However, the clinical outcomes have been met with a mixture of cautious optimism and significant concern. While these treatments can modestly slow the rate of cognitive decline in patients with early-stage AD, the clinical benefits are limited, and their use is accompanied by a substantial risk of serious adverse events, most notably amyloid-related imaging abnormalities (ARIA) (5). This discrepancy between robust plaque clearance and modest clinical efficacy has catalyzed a paradigm shift in the field, compelling a re-evaluation of the linear amyloid cascade and invigorating the search for complementary pathological drivers that must be targeted to achieve meaningful therapeutic impact.

Emerging from the shadow of the amyloid and tau hypotheses, neuroinflammation is now firmly established as a third core pathological pillar of AD (6). This is not a new observation; Alois Alzheimer himself first described the presence of reactive glial cells surrounding senile plaques in 1906 (7). However, for many years, this glial activation was considered a secondary, reactive phenomenon—a mere consequence of the primary proteinopathies (8). A confluence of evidence from large-scale genome-wide association studies (GWAS), epidemiological data, advanced imaging, and neuropathological analyses has overturned this view, repositioning neuroinflammation as a primary, contributing component of AD pathogenesis (9). GWAS have consistently identified variants in immune-related genes, such as Triggering Receptor Expressed on Myeloid Cells 2 (TREM2), Cluster of Differentiation 33 (CD33), and genes within the complement system, as significant risk factors for late-onset AD (LOAD), with many of these genes being predominantly or exclusively expressed in microglia (10, 11). Furthermore, biomarker studies reveal that neuroinflammatory processes are not a late-stage event but are active from the earliest preclinical stages of AD, often preceding clinical onset by years or even decades and co-occurring with the initial deposition of Aβ (12). This early and sustained immune dysregulation is now understood to be a critical driver of the neurodegenerative cascade, making it an indispensable target for early and effective therapeutic intervention.

At the heart of the neuroinflammatory response in AD are microglia, the resident innate immune cells of the central nervous system (CNS) (13). Comprising 5–15% of all cells in the brain, microglia are highly dynamic and multifunctional, acting as sentinels that constantly survey the brain microenvironment (14, 15). In the context of AD, microglia play a complex, multi-stage role that has been recently redefined (16, 17). Contrary to the long-held belief that they are primarily protective in early AD, recent evidence indicates a more complex functional arc (17). In the initial phase, homeostatic microglia act as pathogenic initiators by actively seeding the formation of Aβ plaques, thereby triggering the amyloid cascade (17, 18). As the disease progresses and plaques become established, their role shifts to become protective; activated microglia cluster around deposits to compact them into dense-core plaques (e.g., via TAM receptor engagement), forming a neuroprotective barrier that limits toxicity to surrounding neurons (19–21). However, this protective stance is not indefinite. In the chronic, later stages, relentless stimulation can lead to microglial exhaustion and dysfunction. In this final phase, they can become persistently pro-inflammatory, releasing neurotoxic mediators that damage synapses, promote tau pathology, and contribute to widespread neurodegeneration (13, 22, 23). This intricate and stage-dependent behavior of microglia positions them as a central node in the AD pathological network and, consequently, a critical target for therapeutic modulation.

The growing appreciation for the role of microglia-mediated neuroinflammation in AD has spurred a wave of research into novel therapeutic strategies that move beyond direct Aβ and tau targeting. This review aims to provide a comprehensive and up-to-date analysis of this rapidly evolving field. It will first delineate the dynamic and multifaceted roles of microglia in AD pathogenesis, incorporating recent discoveries that have reshaped our understanding of their function. It will then delve into the core molecular and metabolic mechanisms that drive microglial dysregulation. The central focus of this review will be a critical and structured evaluation of the diverse therapeutic strategies aimed at modulating microglial activity, from suppressing pro-inflammatory responses to enhancing their protective functions and clearing senescent microglial populations. Finally, it will discuss the current clinical trial landscape and the crucial role of biomarkers in translating these innovative approaches into precision medicine for AD.

## 2 The dynamic role of microglia in AD pathogenesis

### 2.1 Microglial heterogeneity and the emergence of the DAM phenotype

The activation states of microglia have traditionally been broadly classified into a simplistic M1/M2 dichotomy, representing classical pro-inflammatory/neurotoxic and alternative anti-inflammatory/neuroprotective phenotypes, respectively. However, this binary classification is now widely recognized as an oversimplification that fails to capture the complex biological roles of microglia in Alzheimer’s disease (AD) pathogenesis (24–27). The M1/M2 concept was originally developed to describe the inflammatory responses of monocyte-derived macrophages. However, as the resident immune cells of the central nervous system (CNS), microglia possess a distinct ontogeny (originating from the yolk sac) and transcriptional features that differ markedly from those of peripheral myeloid cells (28). Importantly, high-resolution analyses have demonstrated that canonical markers of opposing polarization states are often co-expressed within individual microglial cells, and whole-genome expression profiling in multiple mouse models of neurodegeneration has not provided evidence that microglia undergo polarization along a linear M1–M2 axis (28). Therefore, this outdated binary model has been superseded by the understanding that microglial activation is more accurately described as a complex, fluid, and dynamic continuum encompassing multiple functional states, reflecting their response to a chronic, changing CNS microenvironment (25). The advent of powerful high-dimensional technologies, including single-cell RNA sequencing (scRNA-seq), single-nucleus RNA sequencing (snRNA-seq), and single-cell mass cytometry (CyTOF), has been instrumental in revealing the profound heterogeneity and diverse transcriptional and proteomic profiles of microglia, shifting the field’s focus to these nuanced phenotypes (29–31). Such refined molecular characterization is essential, because aging, neurodegeneration, and chronic inflammation drive microglia to undergo functional and phenotypic alterations to varying degrees. Notably, these changes become particularly prominent under chronic inflammatory conditions, which in turn significantly exacerbate neurodegeneration and cognitive decline in AD (31, 32).

One of the most critical discoveries resulting from these advanced transcriptomic analyses is the identification of the Disease-Associated Microglia (DAM) phenotype, a unique microglial type that plays a key role in the pathogenesis of AD and is often found clustered around extracellular Aβ plaques (33). The shift from homeostatic microglia to the DAM state is a tightly regulated, progressive, two-stage activation process, initiated by signals derived from neurodegeneration (34). The initial phase (Stage 1) is recognized as TREM2-independent and is marked by the downregulation of core homeostatic genes such as *P2ry12*, *Cx3cr1*, and *Tmem119* (35). This initial reprogramming sets the stage for the second activation state (Stage 2), which is critically TREM2-dependent and characterized by the robust upregulation of genes involved in phagocytosis, lipid metabolism, and key AD risk factors, including *ApoE*, *Trem2*, *Lpl*, *Cst7*, *Clec7a*, and *Axl* (35, 36). This specific transcriptional signature suggests that DAM cells acquire the functional capacity necessary for the disposal of neurotoxic substances and are highly capable of phagocytosis, actively internalizing both Aβ plaques and pathological tau proteins (33, 34). Interestingly, DAM-like cells exhibiting similar gene signatures have been noted in other neurodegenerative disorders, such as amyotrophic lateral sclerosis (ALS) and multiple sclerosis (MS), suggesting this might represent a generalized immune sensing response to neurodegeneration (36, 37).

Importantly, while the DAM phenotype was primarily characterized in transgenic mouse models, DAM-like transcriptional signatures have also been robustly detected in postmortem human AD brain tissues, including in the original single-cell studies that defined the DAM state (29, 33). This critical overlap bridges the gap between murine models and human pathology. Building upon this translational link, Human Alzheimer’s Microglia (HAM) represent a specialized human population that shows strong transcriptional similarities to murine DAM, particularly concerning genes related to lipid transport and lysosomal function, such as ApoE (38). However, HAM profiles also contain unique upregulated genes not observed in mouse DAM, suggesting critical species-specific differences influenced by manifold environmental and genetic factors (38). Another distinct, and generally detrimental, subtype is the Lipid Droplet-Accumulating Microglia (LDAM), which are characterized by the presence of lipid droplets resulting from metabolic dysfunctions and neuroinflammation in the aging brain (39). This accumulation of lipid droplets contributes to increased oxidative stress and severely impairs the microglia’s ability to clear aggregates through phagocytosis, thereby representing a dysfunctional and proinflammatory state that drives AD progression (40, 41). Relatedly, the Microglial Neurodegenerative Phenotype (MGnD) is also recognized, observed mainly in the terminal stages of neurodegeneration in AD models (like APP-PS1), where TREM2 signaling is again essential for driving this transition away from the homeostatic state (42). This expanding knowledge of distinct states—including Interferon Response Microglia (IRM), Activated Response Microglia (ARM), and Cycling/proliferating microglia (CPM)—reinforces the recognition that microglial activity is not simply “activated” but highly diverse, necessitating a tailored approach to therapeutic intervention (43, 44).

Understanding microglial states as a rich and variegated landscape, rather than a fixed dichotomy, offers a powerful framework for developing precision therapeutic strategies. Since microglia act as a “double-edged sword” with both protective and detrimental effects, future success in AD treatment relies on identifying and selectively modulating these specific, disease-relevant phenotypes, such as enhancing protective phagocytosis functions (e.g., through TREM2 activation) while inhibiting the deleterious pro-inflammatory aspects driven by subsets like LDAM. Just as a skilled artisan must use a full spectrum of specialized tools rather than relying on only two generic ones, researchers must leverage the discovery of these microglial subsets to precisely tune the immune environment for neuroprotection.

### 2.2 Stage-dependent functional transitions

The functional identity and pathological contribution of microglia are fundamentally dictated by the temporal evolution of AD pathology (17). Rather than assuming a static, unitary role, current evidence supports that microglia transition through distinct, often contradictory, functional states along the disease trajectory (16). This dynamic role spans from the clinically silent pre-amyloid stage to the devastating neuroinflammation characteristic of advanced dementia.

Historically, descriptive studies in transgenic AD models, such as the triple transgenic (3xTg-AD) mouse model, revealed that an increase in the density of resting microglia precedes the formation of neuritic plaques and microglial activation (45). This observation led to the interpretation that microglia, being the intrinsic CNS defense system, were proliferating “as if in preparation for the ensuing activation” required to combat the extracellular Aβ load characteristic of terminal disease stages (45). However, employing methods such as Colony-Stimulating Factor 1 Receptor (CSF1R) inhibitors (e.g., PLX3397 or PLX5622) for microglial depletion prior to Aβ deposition has recently shifted this paradigm dramatically (17). New evidence suggests that homeostatic microglia—the pre-activated phenotype—actively facilitate the initiation (seeding) of amyloid plaques (17, 18). When homeostatic microglia were ablated early in the disease course, a resulting reduction in the overall number of plaques and associated neuritic dystrophy was observed (17, 46). This finding establishes that microglia operate upstream of Aβ deposition, challenging the traditional view that they are merely reactive to established plaques. Further refining this, functional studies revealed that while early continuous depletion significantly reduced insoluble Aβ levels (plaque burden), the concentration of soluble Aβ remained remarkably consistent (46, 47). This specific divergence strongly questions the conventional assumption that the primary role of microglia is efficient Aβ clearance during these initial preclinical stages, highlighting their role in plaque assembly instead (46). Moreover, early alterations involving this microglial proliferation, often coinciding with astrocyte atrophy, may represent a pathologically relevant step linked to the cognitive decline observed in the early stages of AD (45).

**Figure 1:**
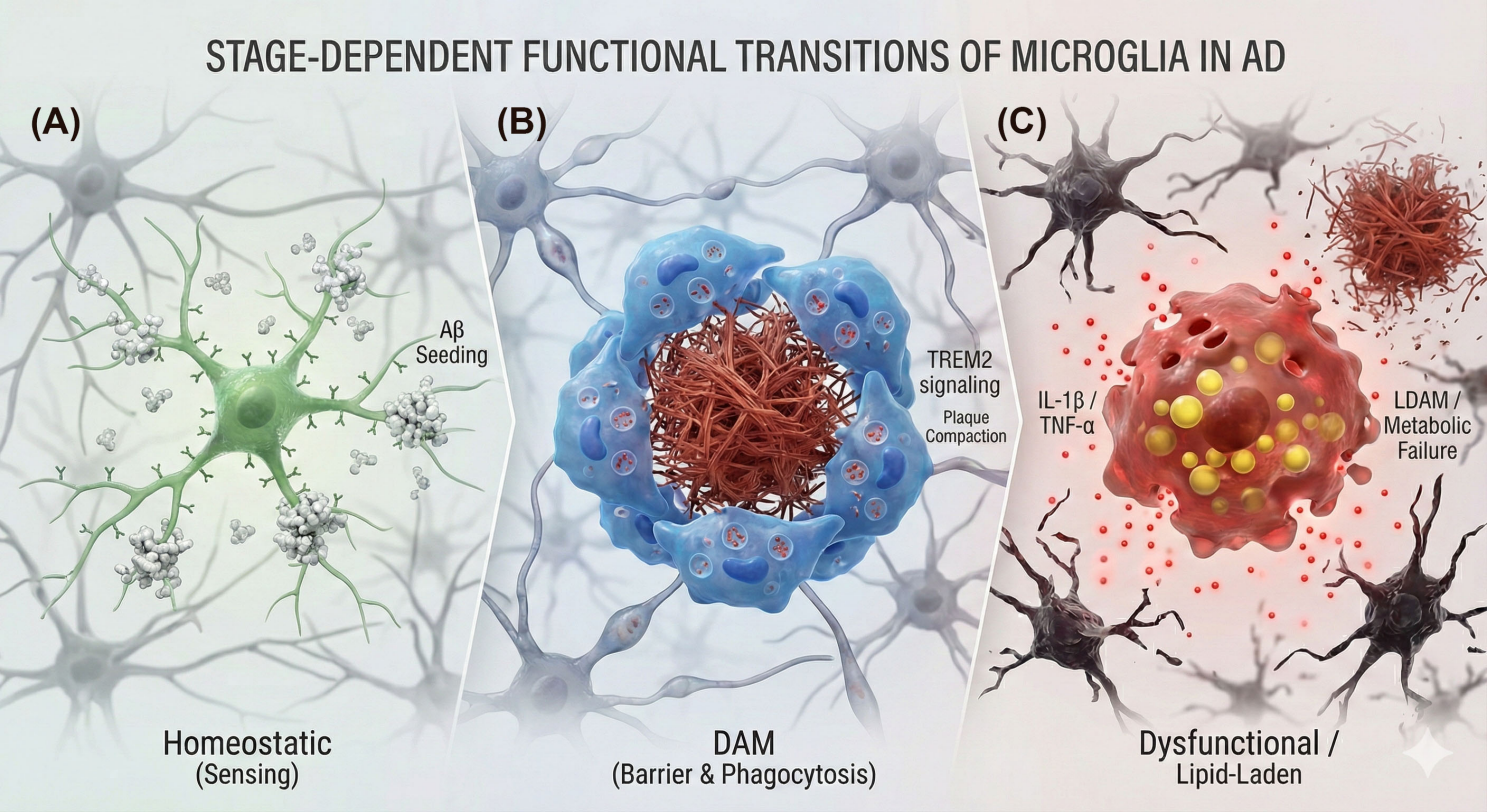
Stage-dependent functional transitions of microglia in Alzheimer’s disease (AD) pathogenesis. This schematic illustrates the dynamic and distinct functional phases of microglia throughout the trajectory of AD,highlighting their “double-edged sword” nature. In the initial preclinical phase **(A)**,homeostatic microglia actively survey the central nervous system (CNS) microenvironment; however,contrary to a purely protective role,they may inadvertently facilitate the initial seeding of Aβ plaques upstream of overt pathology. As the disease progresses to the amyloid deposition phase **(B)**,microglia undergo a Triggering Receptor Expressed on Myeloid Cells 2 (TREM2)-dependent transcriptional reprogramming to acquire the Disease-Associated Microglia (DAM) phenotype. These activated cells cluster around Aβ deposits to compact them into dense-core plaques,effectively forming a neuroprotective barrier that limits the diffusion of neurotoxic species and protects surrounding neurites. Conversely, in the chronic and late stages of the disease **(C)**, relentless pathological stimulation leads to microglial exhaustion and metabolic failure,typified by the Lipid Droplet-Accumulating Microglia (LDAM) phenotype. In this dysfunctional state,microglia lose their phagocytic barrier capacity and become a primary source of chronic neuroinflammation,releasing cytotoxic pro-inflammatory mediators such as IL-1β and TNF-α via NLRP3 inflammasome activation,thereby driving widespread synaptic loss and neurodegeneration.

As Aβ deposition becomes consolidated and the disease progresses into the mid and late phases, microglia undergo a phase of rapid and massive activation, transitioning into highly plastic and heterogenous states, such as the DAM phenotype (45, 48). This state involves the sequential upregulation of genes critical for lipid metabolism and phagocytosis, particularly within the TREM2-APOE pathway (36). In this later context, activated microglia fulfill a crucial protective and remodeling role by aggregating closely around mature Aβ plaques, a process deeply reliant on specific phagocytic receptors such as the TAM family (9, 19–21). This response leads to plaque compaction, limiting the diffusion of neurotoxic Aβ species and significantly reducing the associated neuritic dystrophy. The loss-of-function variants in genes like *Trem2* impair this critical barrier function, leading to more diffuse plaques and severe axonal dystrophy (49). However, this period of activation is a “double-edged sword”: chronic, sustained activation leads to a maladaptive neurotoxic state (16, 50). Activated microglia are the primary source of chronic neuroinflammation, releasing a cytotoxic cocktail of pro-inflammatory factors (such as IL-1β, IL-6, and TNF-α) (49, 51). This chronic inflammatory environment causes neuronal damage, exacerbates synaptic loss, and promotes the activation of the NLRP3 inflammasome (9). The activation of NLRP3 not only drives inflammation but can also lead to the release of ASC (an apoptosis-associated speck-like protein containing a caspase recruitment domain) specks, which further seed Aβ deposition and drives tau pathology. Concurrently, microglial activation, often associated with systemic inflammatory factors, contributes to the hyperphosphorylation and spreading of pathological tau throughout the brain (52, 53). In the terminal stages, microglia may become functionally impaired, characterized by dystrophic (senescent) morphology and declining phagocytic capacity toward Aβ aggregates, thereby accelerating neurodegeneration (**Figure 1**) (54).

The intricate, stage-dependent functional switch—from early amyloid seeders to protective plaque compactors that concurrently drive chronic neurotoxicity—presents significant challenges for therapeutic strategies. The traditional approach of reducing inflammation using non-steroidal anti-inflammatory drugs (NSAIDs) may be ineffective or even detrimental if timed incorrectly (8). Furthermore, therapeutic attempts that aim to enhance protective functions, such as those employing TREM2 agonists to boost phagocytosis, may face limitations in the advanced disease stages due to existing microglial dysfunction or exhaustion (55). Conversely, microglial depletion strategies, such as using CSF1R inhibitors, show promise in the early stages by preventing plaque seeding, but continuous treatment could potentially hinder the later, beneficial compaction role of activated microglia (17). Therefore, achieving success in AD therapy necessitates a comprehensive understanding of microglial functional changes and roles, focusing on precise, stage-specific modulation of microglial activity and phenotype to enhance beneficial functions while mitigating the detrimental chronic neuroinflammation.

### 2.3 Crosstalk between microglia, Aβ, Tau, and astrocytes

Microglia are central orchestrators of the pathological crosstalk in AD, engaging in a complex and dynamic interplay with Aβ, tau, and astrocytes (54). This network of interactions forms self-perpetuating feedback loops that transform the neuroinflammatory response from a protective mechanism into a core driver of neurodegeneration (56). Understanding this intricate crosstalk is fundamental to developing effective therapeutic strategies that can modulate microglial function.

The interaction with Aβ initiates this pathological cascade and exemplifies the dual nature of microglia (57). Through an array of surface pattern recognition receptors (PRRs) like TREM2 and Toll-like receptors (TLRs), microglia recognize and attempt to clear Aβ deposits via phagocytosis (58–60). However, this same engagement, particularly through receptors like TLRs and CD36, simultaneously triggers potent intracellular inflammatory pathways, notably NF-κB and the NLRP3 inflammasome (54, 61). The resulting chronic production of pro-inflammatory cytokines, such as TNF-α and IL-1β, not only causes direct neurotoxicity but also paradoxically increases Aβ production by upregulating β- and γ-secretase expression (62, 63). Furthermore, NLRP3 inflammasome activation leads to the release of ASC specks, which act as extracellular seeds to accelerate Aβ aggregation, thus establishing a destructive feed-forward loop (58).

This Aβ-driven microglial activation serves as a critical upstream driver that links the two core proteinopathies of AD (Aβ and tau) (8). Activated microglia play a similarly dual role in tau pathology, attempting to clear pathological tau while also contributing to its prion-like propagation through the release of tau-containing exosomes (54, 64–66). Crucially, the inflammatory environment established by the Aβ-microglia interaction directly exacerbates tau pathology (8). Inflammatory mediators like IL-1β, produced via NLRP3 activation, can promote tau hyperphosphorylation and aggregation (9). This positions microglia as the key cellular mediator through which the amyloid cascade potentiates the spread of tauopathy, directly connecting the two pathological hallmarks through a shared inflammatory mechanism.

The neuroinflammatory landscape is further amplified by a detrimental crosstalk between microglia and astrocytes (61). As fundamentally demonstrated by Liddelow et al., activated microglia release a cocktail of inflammatory factors, including TNF-α, IL-1α, and C1q, which induce a profound phenotypic shift in neighboring astrocytes, converting them into a neurotoxic “A1” state (67). These A1 astrocytes abandon their crucial neurosupportive functions and instead release factors that actively destroy synapses and neurons (61, 67). This microglia-astrocyte axis establishes a powerful mechanism for amplifying and sustaining the neurotoxic environment, pushing the brain further into a state of chronic, non-resolving inflammation and widespread cellular damage.

**Figure 2:**
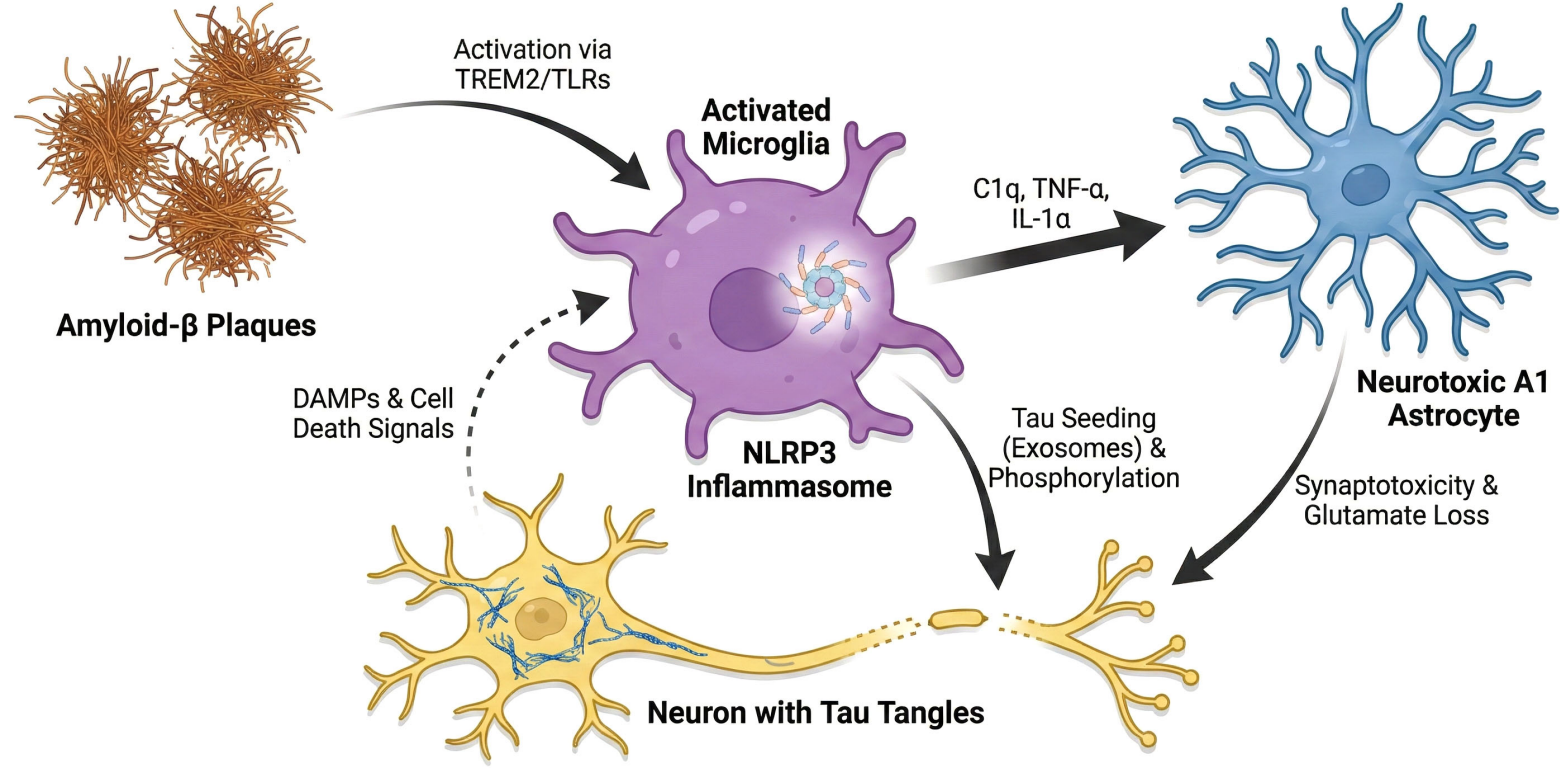
The vicious cycle of microglia-mediated neurotoxicity and multicellular crosstalk in AD. This diagram illustrates how activated microglia (center,purple) act as the central node driving a self-perpetuating neurodegenerative loop. **(A)** The cycle initiates when microglia recognize Aβ plaques via receptors such as TREM2 and Toll-like receptors (TLRs),leading to the activation of the NLRP3 inflammasome and the release of pro-inflammatory mediators. **(B)** Microglia secrete a specific cocktail of cytokines—C1q,TNF-α,and IL-1α—which induces a phenotypic shift in astrocytes,converting them into neurotoxic A1 astrocytes. these A1 astrocytes lose neuroprotective functions and actively promote synaptic destruction. **(C)** Concurrently,microglial inflammation exacerbates tau pathology by promoting hyperphosphorylation (via kinase activation) and facilitating the spreading of tau seeds through exosomal transport. **(D)** The combined toxicity leads to neuronal death and the release of damage-associated molecular patterns (DAMPs),which provide a feedback signal to further activate microglia,thereby sustaining chronic neuroinflammation and accelerating disease progression.

Given this intricate pathological interplay, where microglia act as a central node driving a self-sustaining cycle of neurotoxicity, it is clear why therapeutic approaches must evolve beyond simply targeting Aβ or tau in isolation (**Figure 2**). Instead, the focus must shift to precisely modulating the microglial response: enhancing their beneficial phagocytic functions, potentially through TREM2 agonists, while simultaneously suppressing the chronic, detrimental inflammatory signaling driven by pathways like the NLRP3 inflammasome and NF-κB. The ultimate goal is to disrupt these pathological feedback loops, shifting microglia from a neurotoxic state back to a homeostatic, neuroprotective one. Success will likely depend on precision-based combination therapies that can restore balance to this complex cellular network, thereby halting the progression of the disease.

## 3 Therapeutic strategies targeting microglial regulation

The therapeutic landscape of AD is currently witnessing a paradigm shift, transitioning from a purely neurocentric and amyloid-centric view toward a more holistic understanding that places the innate immune system at the core of disease pathogenesis. Microglia, the resident immune sentinels of the CNS, act as a double-edged sword in the progression of AD. In their homeostatic state, they are essential for the phagocytic clearance of neurotoxic debris, synaptic pruning, and the maintenance of a healthy neural environment. However, chronic activation of these cells by pathological protein aggregates—such as Aβ and tau—drives a self-perpetuating cycle of neuroinflammation, synaptic toxicity, and metabolic exhaustion. This maladaptive state, often referred to as DAM or MGnD, represents a distinct cellular phase of AD that offers a wealth of novel therapeutic targets. Consequently, emerging therapeutic strategies are categorized not merely by their molecular targets, but by their functional intent: enhancing protective clearance mechanisms, arresting neurotoxic inflammatory signaling, restoring metabolic plasticity, eliminating senescent cell populations, and optimizing delivery and efficacy via advanced nanotechnology and combinatorial approaches. This section provides an exhaustive analysis of these strategies, synthesizing preclinical mechanistic insights with the latest clinical trial data to delineate the current state and future direction of microglia-targeted therapies.

### 3.1 Modulating microglial clearance and phagocytic receptors

**Figure 3:**
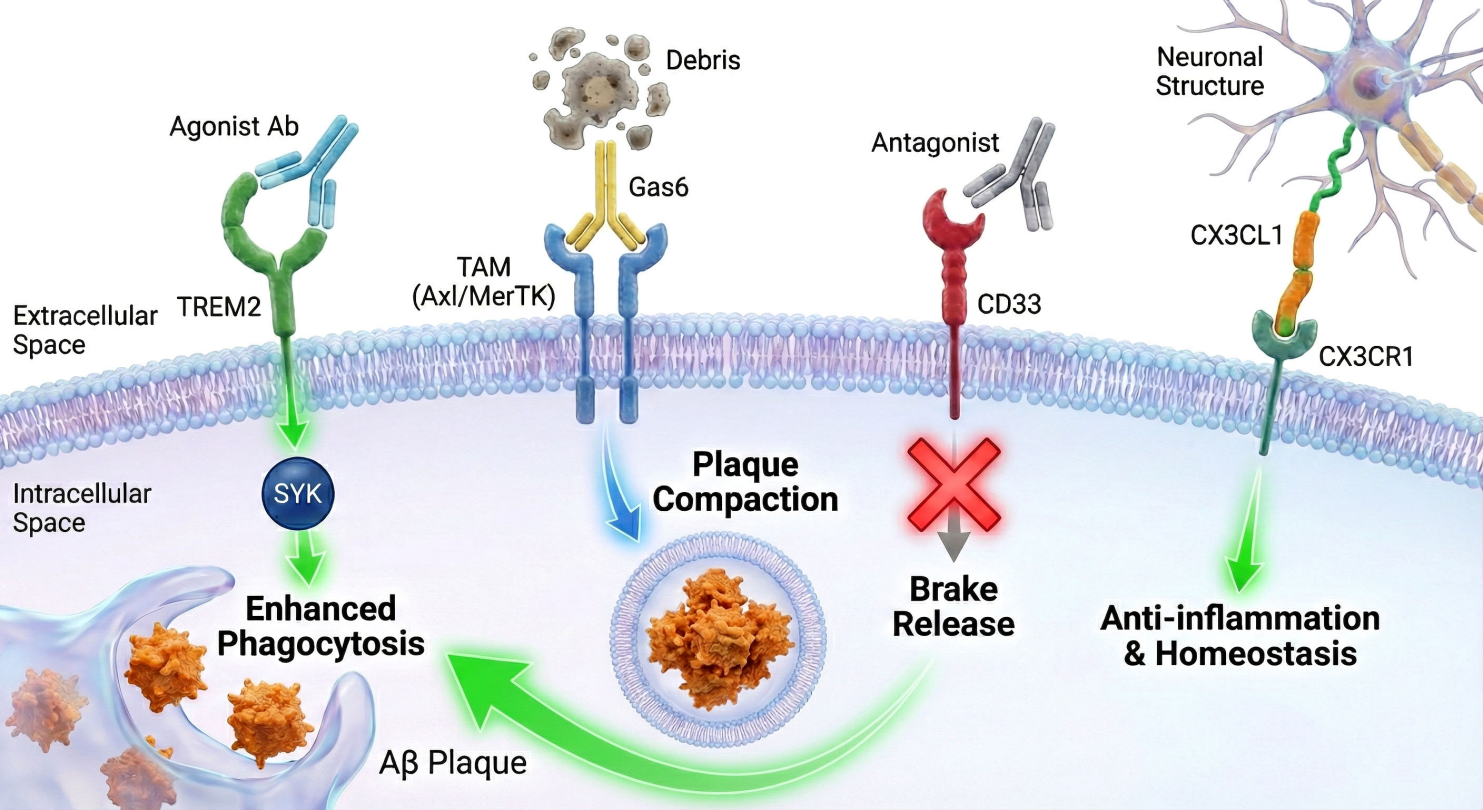
Therapeutic strategies targeting microglial surface receptors to modulate clearance and homeostasis. The schematic illustrates four pivotal receptor axes on the microglial membrane (purple) regulating functional transitions. **(A)** Agonism of TREM2 using specific antibodies (Agonist Ab) activates downstream SYK signaling,promoting the transformation into a responsive state that drives the enhanced phagocytosis of Aβ plaques. **(B)** The TAM family receptors (Axl/MerTK) engage with apoptotic debris via the bridging ligand Gas6,facilitating efferocytosis and plaque compaction. **(C)** The CD33 acts as an inhibitory checkpoint; therapeutic blockade with an antagonist prevents downstream inhibitory signaling (Red X),effectively resulting in a “brake release” to restore phagocytic competency. **(D)** The CX3CR1 receptor binds to neuronal CX3CL1 (fractalkine),maintaining a neuroprotective checkpoint that fosters anti-inflammation and homeostasis. Abbreviations: Aβ,amyloid-beta; SYK,spleen tyrosine kinase; Gas6,growth arrest-specific 6; TAM,Tyro3,Axl,and MerTK family; TREM2,triggering receptor expressed on myeloid cells 2; CD33,cluster of differentiation 33.

AD pathogenesis is accelerated by the failure of microglia to effectively sense, engulf, and degrade pathological substrates (68). Genetic association studies have been pivotal in identifying risk variants in surface receptors that regulate these functions (69). The most prominent among these are the TREM2, the sialic acid-binding immunoglobulin-like lectin 3 (CD33), the TAM receptor family (Tyro3, Axl, MerTK), and the CX3CL1-CX3CR1 checkpoint axis. Therapeutic strategies in this domain aim to selectively activate protective sensors or antagonize inhibitory checkpoints to restore microglial competency and re-enable the clearance of Aβ and cellular debris (**Figure 3**).

#### 3.1.1 Promoting the neuroprotective DAM state via TREM2 agonism

TREM2, a type I transmembrane receptor predominantly found on microglia within the CNS, stands as arguably the most critical genetic and therapeutic target in AD pathogenesis. Genetic variants, notably the R47H mutation, significantly elevate the risk of LOAD, acting primarily as loss-of-function mutations that impair microglial defense mechanisms (70). TREM2 is indispensable for numerous essential microglial functions, including survival, proliferation, chemotaxis, phagocytosis, and maintaining lipid metabolic fitness (71). Mechanistically, TREM2 engagement is crucial for driving the transition from homeostatic microglia to the protective DAM phenotype (specifically Stage 2 activation). This state acquires robust phagocytic capabilities essential for engulfing pathological proteins like Aβ and tau, thereby restricting plaque seeding and spread. This signaling axis also regulates inflammation and microglial phenotype shift via pathways including SYK and PI3K/AKT/mTOR cascades (57). Consequently, therapeutic agonism aimed at boosting TREM2 signaling constitutes a major strategy to enhance phagocytosis, clear pathology, and restore neuroimmune homeostasis.

This therapeutic direction has fueled the intensive development of agonistic mAbs. Leading candidates such as AL002 (including variants AL002a/c), Ab-T1, Ab18 and 4D9 have been investigated to enhance TREM2 function (72). These agonistic antibodies typically function by promoting receptor cross-linking to amplify the downstream DAP12/SYK signaling cascade, consequently boosting microglial proliferation and phagocytic activity. A related strategy employed by antibodies like AL002c and 4D9 involves binding near the ADAM10/ADAM17 cleavage site on the extracellular stalk region. This binding inhibits ectodomain shedding, thereby stabilizing full-length TREM2 on the cell surface and terminating the generation of soluble TREM2 (sTREM2). Preclinical studies demonstrated that these TREM2 agonists reduced plaque burden and ameliorated cognitive deficits in various AD mouse models by promoting migration and phagocytosis. However, the recent Phase II INVOKE-2 trial evaluating AL002 (NCT04592874) did not meet its primary endpoints, failing to significantly slow clinical progression or reduce amyloid burden, despite achieving target engagement (73). This outcome, coupled with preclinical findings that chronic TREM2 activation can potentially exacerbate Aβ-associated tau seeding and spreading, underscores the complexities and context-dependent nature of TREM2-targeted therapy.

To circumvent the limitations associated with large therapeutic biologics, particularly poor blood-brain barrier (BBB) penetration, researchers are actively developing brain-penetrant small-molecule TREM2 agonists. VG-3927 (Vigil Neurosciences) represents a key small-molecule candidate currently undergoing Phase I clinical evaluation (NCT06343636) (56). This orally bioavailable compound is notable for functioning as a molecular glue, selectively potentiating the function of membrane-bound TREM2 in response to natural ligands without directly binding to the decoy sTREM2 fragment (74). Initial pharmacokinetic/pharmacodynamic (PK/PD) studies for VG-3927 demonstrated brain penetrance and pharmacological activity in the CNS (75). Beyond synthetic compounds, preclinical data also supports the potential of natural compounds, such as the plant alkaloid Hecubine and extracts from Pyrolae Herba, which directly activate TREM2 signaling and mitigate neuroinflammation (76). Ultimately, optimizing the precise therapeutic window for TREM2 agonism—likely initiated during early, protective pathological stages—is crucial to maximize protective phagocytic clearance while mitigating the risk of inadvertent pro-inflammatory or detrimental activation observed later in the disease course. Crucially, recent breakthrough evidence provides a mechanistic explanation for this detrimental late-stage activation, revealing that chronic engagement of the TREM2-APOE signaling axis can paradoxically drive microglia into a dysfunctional, senescent state. This suggests that sustained TREM2 activation over time may shift the microglial phenotype from protective clearance to senescence-associated chronic inflammation (77).

#### 3.1.2 Unleashing phagocytic capacity through CD33 inhibition

Modulating microglial function by targeting inhibitory surface receptors provides a critical strategy complementary to promoting activation, aimed specifically at engaging clearance mechanisms by lifting the physiological “brake” imposed by CD33. CD33, a sialic acid-binding immunoglobulin-like lectin (Siglec-3), is encoded by a key AD susceptibility gene and is predominantly expressed on microglia and other myeloid cells (78). Its inhibitory function stems from its intracellular Immunoreceptor Tyrosine-based Inhibition Motif (ITIM) domain, which, upon phosphorylation, recruits phosphatases that suppress core microglial activities such as phagocytosis and proliferation (79). This mechanism is highly relevant to AD pathology, as elevated CD33 expression correlates strongly with impaired Aβ clearance and increased plaque burden (80). Conversely, genetic evidence supports the benefit of counteracting this brake: individuals carrying protective *CD33* alleles exhibit reduced functional expression and a significantly lower risk of developing AD (80).

Consequently, inhibition of CD33 represents a compelling therapeutic avenue to restore microglial phagocytic capacity. Preclinical models validate this approach, demonstrating that *CD33* knockout significantly enhances microglial phagocytosis of Aβ aggregates, resulting in reduced amyloid pathology and improved cognitive function in 5xFAD mice (78). Critically, emerging evidence suggests that the protective effect of relieving the CD33 brake is contingent upon the presence of functional TREM2 signaling (78). This establishes that TREM2 acts downstream of CD33 in modulating microglial pathology, indicating that the therapeutic benefit of CD33 inhibition can only be fully realized when the TREM2-dependent clearance machinery is intact. Therapeutic candidates pursuing this strategy include small-molecule inhibitors, such as the sialic acid mimetic P22, and antagonistic mAbs like AL003 and Lintuzumab (81). While trials for agents like AL003 have highlighted the challenges of clinical translation, the strategy of blocking CD33 activity to unleash microglial clearance remains a priority in drug discovery.

#### 3.1.3 The role of TAM receptors in efferocytosis and plaque compaction

The TAM receptor family—comprising Tyro3, Axl, and MerTK—orchestrates a critical, yet distinct, axis of microglial regulation focused on efferocytosis and the clearance of membrane debris. Unlike the constitutively expressed receptors involved in general surveillance, the TAM family exhibits a distinct expression shift during AD progression: MerTK is predominant in homeostatic microglia and mediates the immunologically “silent” clearance of apoptotic cells, whereas Axl is normally quiescent but undergoes dramatic upregulation in plaque-associated microglia (82). These receptors function through a unique bridging mechanism, binding to ligands such as growth arrest-specific 6 (Gas6) and Protein S (Pros1), which in turn recognize “eat-me” signals like exposed phosphatidylserine (PtdSer) on the surface of apoptotic neurons or amyloid aggregates (21).

The contribution of TAM receptors to AD pathology is characterized by a complex functional dichotomy that challenges straightforward therapeutic intervention. Mechanistically, TAM signaling is indispensable for the recognition and engulfment of amyloid plaques. Studies employing Axl/MerTK double-knockout models have revealed that the loss of this signaling pathway results in a failure of microglia to engage with plaques, leading to the accumulation of uncompacted, “fluffy” amyloid deposits and severe dystrophic neurites (21). This evidence suggests that TAM-driven phagocytosis is essential for plaque compaction—a process that creates a physical barrier to contain neurotoxicity. However, this protective function presents a paradox: the very machinery that contains toxicity also promotes the consolidation of dense-core plaques. While these dense plaques are generally considered less neurotoxic than diffuse oligomers, they remain a pathological hallmark, raising the question of whether therapeutic modulation should aim to inhibit plaque formation or enhance compaction.

Emerging evidence provides a compelling consensus to this question: therapeutic interventions should aim to preserve or augment, rather than suppress, TAM-mediated plaque compaction. The initial hypothesis—suggesting that inhibiting upregulated Axl could beneficially mitigate plaque-associated inflammation—has been substantially challenged by recent *in vivo* models. Given that the abrogation of TAM signaling actively exacerbates neuritic dystrophy and neurotoxicity, the therapeutic paradigm is currently pivoting from broad receptor antagonism toward precision enhancement and targeted modulation.

One promising strategy involves stabilizing the full-length receptors on the microglial surface. During disease progression, metalloproteases such as ADAM10 and ADAM17 cleave Axl and MerTK into soluble forms (e.g., sAxl), which act as decoy receptors that sequester endogenous ligands like Gas6, thereby interrupting the protective signaling cascade (83). Inhibiting these metalloproteases could theoretically restore microglial phagocytic capacity. Furthermore, emerging preclinical evidence highlights the potential of small molecules to therapeutically engage this system. For instance, Ganoderic acid A has been shown to relieve Aβ burden by enhancing Axl-mediated autophagy, and Jujuboside A promotes Aβ clearance via the Axl/PPARγ axis (84, 85). Ultimately, both interventions ameliorate cognitive deficits in animal models.

Perhaps the most innovative translational approach utilizes the TAM system to decouple Aβ clearance from neurotoxic inflammation. Recent engineered biologics have successfully fused single-chain anti-Aβ antibodies to the TAM-receptor-binding domain of Gas6. This novel chimeric strategy selectively directs Aβ to TAM receptors, inducing robust microglial phagocytosis without triggering the classical inflammatory response or risking cerebral amyloid angiopathy (86). Consequently, future therapeutic strategies targeting the TAM family must move beyond simplistic agonism or antagonism. Success will rely on sophisticated interventions that harness MerTK and Axl to facilitate the immunologically “silent” clearance of pathological fibrils, thereby restoring neuroimmune homeostasis without collateral inflammatory damage.

#### 3.1.4 Modulating the CX3CL1-CX3CR1 neuron-microglia checkpoint

The CX3CL1-CX3CR1 axis serves as a fundamental homeostatic checkpoint for neuron-microglia communication, distinguished by its high-affinity, one-to-one fidelity in the CNS (87). In the healthy brain, neuronal CX3CL1 (Fractalkine) binds to microglial CX3CR1, functioning as a constitutive “calming signal” that inhibits pro-inflammatory pathways, such as NF-κB, and maintains microglia in a surveillance state (88). Emerging mechanistic insights have revealed that this interaction is bidirectional: beyond the “forward signaling” that regulates microglial phagocytosis and oxidative stress via Nrf2, a “back-signaling” mechanism exists where the intracellular domain (ICD) of CX3CL1 translocates to the neuronal nucleus, promoting cell survival and neurogenesis through TGF-β/Smad pathways (89, 90).

In the context of Alzheimer’s pathology, this axis presents a complex “Janus-faced” paradox. Genetic deficiency or disruption of CX3CR1 signaling acts as a “release of the brake,” which facilitates the phagocytic clearance of fibrillar amyloid plaques; however, this disinhibition inadvertently increases the accumulation of neurotoxic soluble oligomers and exacerbates synaptic loss (91, 92). Crucially, the integrity of this axis is unequivocally protective against tau pathology. Disruption of signaling amplifies a p38 MAPK-IL-1β inflammatory loop that drives GSK3β-mediated tau hyperphosphorylation, while simultaneously impairing the microglial capacity to internalize and degrade extracellular tau seeds (53, 93, 94).

Given these divergent roles, therapeutic modulation requires a precise approach that avoids the pitfalls of broad antagonism. While CX3CR1 antagonists may reduce peripheral inflammation, they risk accelerating tau propagation and cognitive decline within the CNS (95). Consequently, the most promising strategies involve agonism to restore this neuroprotective “handshake.” Interventions utilizing viral vectors to deliver soluble Fractalkine (sFKN) or its C-terminal fragment have demonstrated efficacy in reducing tau pathology and preventing neurodegeneration without compromising amyloid clearance mechanisms (89, 96, 97). Future development aims to identify small-molecule agonists or gene therapies that can selectively boost these signaling pathways to decouple neuroinflammation from neurodegeneration.

### 3.2 Blocking neurotoxic inflammation and downstream signaling

**Figure 4:**
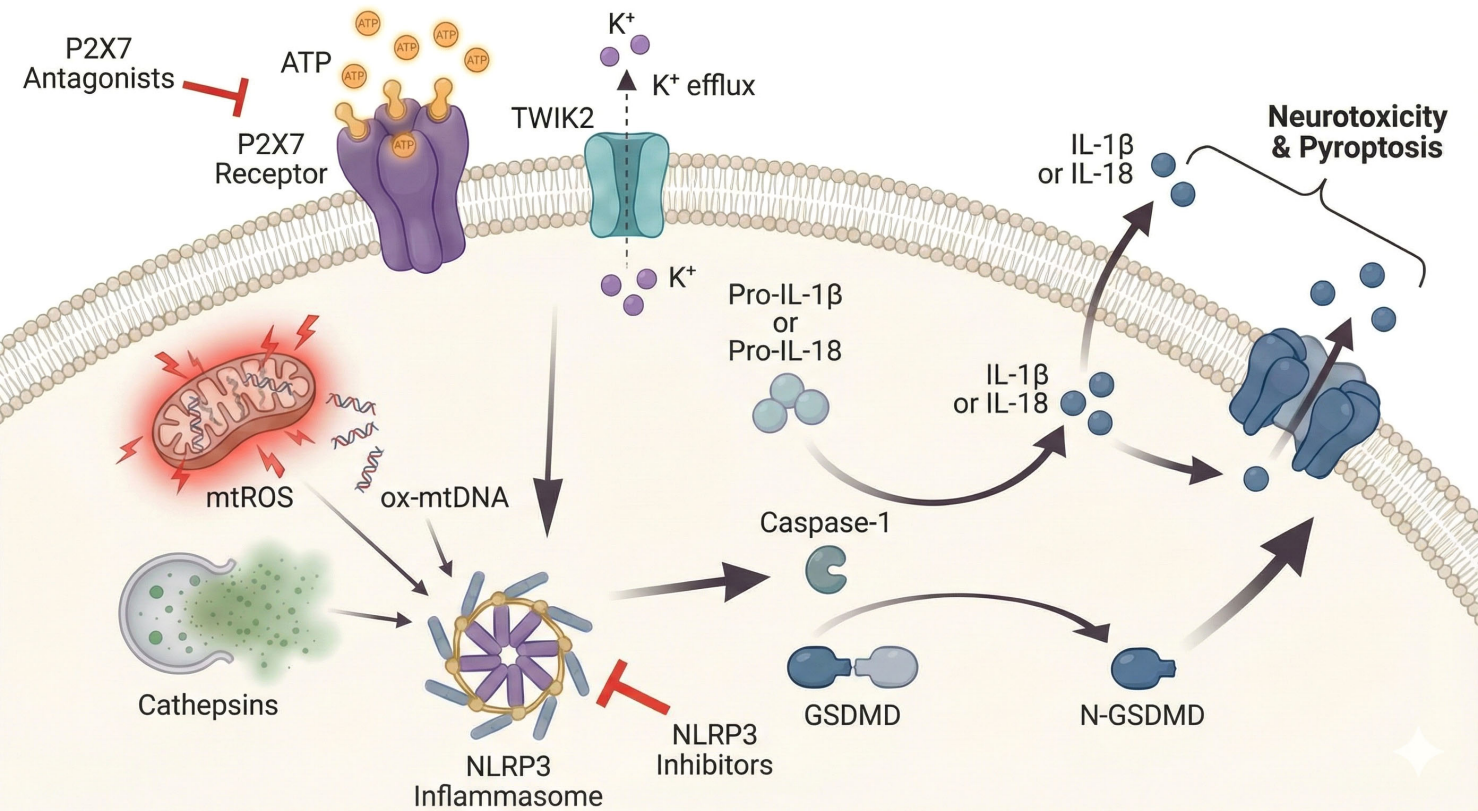
Schematic representation of the P2X7-NLRP3 inflammasome signaling cascade and therapeutic targets in microglia. Pathological stimuli,such as high concentrations of extracellular ATP (eATP),activate the P2X7 receptor (P2X7R),triggering potassium (K + +) efflux (facilitated by the TWIK2 channel). This ionic imbalance,combined with intracellular stress signals including mitochondrial dysfunction (mtROS and oxidized mtDNA) and lysosomal destabilization (Cathepsins),drives the assembly and activation of the NLRP3 inflammasome. Once active,the inflammasome promotes the maturation of Caspase-1,which subsequently cleaves pro-IL-1β and pro-IL-18 into their active forms. Caspase-1 also cleaves Gasdermin D (GSDMD),liberating the N-terminal domain to form non-selective pores in the plasma membrane. These pores facilitate the release of inflammatory cytokines and induce pyroptosis,contributing to neurotoxicity. Potential therapeutic strategies are highlighted by red inhibition bars: P2X7 antagonists block the upstream activation trigger,while specific NLRP3 inhibitors prevent inflammasome assembly,effectively arresting the inflammatory cascade.

While enhancing microglial clearance is a primary therapeutic goal, it is equally critical to arrest the maladaptive inflammatory cascades that perpetuate neurodegeneration. In AD, the chronic engagement of PRRs not only fails to resolve pathology but also triggers a sustained release of neurotoxic mediators that damages neurons and disrupts the BBB. Therefore, distinct therapeutic strategies must be employed to sever the link between pathological recognition and the release of neurotoxic mediators. This section focuses on interventions targeting the core machinery of this response—specifically the NLRP3 inflammasome and its upstream trigger, the P2X7 receptor (P2X7R)—to prevent the release of downstream cytokine executioners (**Figure 4**).

#### 3.2.1 The NLRP3 inflammasome as a central driver of neuroinflammation

The NLRP3 inflammasome acts as a critical convergence point for the multiple pathological insults characterizing the AD brain, functioning not merely as a responder to pathology but as an active generator of it. Structurally, this cytosolic complex consists of the sensor molecule NLRP3, the adaptor protein ASC, and the effector pro-caspase-1. Its activation in microglia is tightly regulated by a two-step mechanism comprising priming and activation. In the AD context, the initial priming signal triggers NF-κB-mediated transcriptional upregulation of NLRP3 and pro-IL-1β via Aβ interaction with PRRs (98, 99). While this prepares the cell, the catalytic trigger arises from metabolic and physical stress. Specifically, the “frustrated phagocytosis” of fibrillar Aβ destabilizes lysosomes, releasing cathepsin B, while P2X7R activation by extracellular ATP (eATP) drives potassium efflux. Simultaneously, mitochondrial dysfunction generates reactive oxygen species (mtROS) and releases oxidized mitochondrial DNA (mtDNA), which are sensed by the NACHT domain of NLRP3, initiating rapid complex assembly (100, 101).

Once assembled, the inflammasome drives pathology through unique downstream mechanisms, most notably via the formation of ASC specks. Released into the extracellular space following pyroptosis, these specks exhibit prion-like bioactivity. Driven by specific electrostatic interactions and molecular mimicry, the ASC pyrin domain (PYD) binds Aβ, significantly accelerating its aggregation and spreading plaque deposition in a cross-seeding manner (102, 103). Furthermore, NLRP3 activation creates a feed-forward loop with tau pathology. The release of IL-1β enhances the activity of tau kinases (e.g., GSK-3β) while suppressing phosphatases. Recent evidence further indicates that tau monomers can activate NLRP3, which reciprocally promotes tau acetylation and aggregation, thereby driving neurofibrillary tangle formation and hippocampal atrophy (104).

The terminal output of this cascade is pyroptosis, a lytic form of cell death mediated by Gasdermin D (GSDMD). Active caspase-1 cleaves GSDMD, liberating its N-terminal domain to form large non-selective pores in the plasma membrane. These pores serve as the necessary conduit for the release of cytokines IL-1β and IL-18, which lack secretory signal peptides, fueling a cytokine storm (105, 106). Beyond cytokine release, GSDMD pore formation compromises the integrity of microglia and, as recent high-resolution studies suggest, surrounding neurons. The resulting loss of synaptic integrity due to GSDMD cleavage directly links the inflammatory machinery of the inflammasome to the neurodegeneration and cognitive decline observed in AD (107).

#### 3.2.2 Advances in small molecule NLRP3 inhibitors

Targeting the NLRP3 inflammasome offers a precision medicine approach that addresses both the seeding of proteinopathies and the neurotoxic inflammatory response while sparing other innate immune pathways essential for host defense. The prototype for this strategy, MCC950 (CRID3), specifically binds to the Walker B motif of the NLRP3 NACHT domain, locking the protein in an inactive conformation (108, 109). While MCC950 successfully reversed cognitive deficits and altered microglial phenotypes in APP/PS1 mice, its development was halted due to hepatotoxicity and limited BBB penetrance (110, 111). Nevertheless, it paved the way for safer candidates like OLT1177 (Dapansutrile) and CY-09. OLT1177 inhibits NLRP3 ATPase activity without affecting transcriptional priming and has demonstrated efficacy in restoring synaptic plasticity in AD models (112). Similarly, CY-09 targets the ATP-binding motif to block assembly and restore cerebral glucose metabolism, while the repurposed anti-allergic drug Tranilast offers a safe, immediate option for inhibiting NLRP3 oligomerization (113).

The field is now advancing towards “next-generation” inhibitors explicitly optimized for CNS pharmacokinetics and potency. Leading this wave is NT-0796, which utilizes an innovative ester prodrug mechanism. Being lipophilic, NT-0796 efficiently crosses the BBB and cell membranes, where intracellular esterases convert it into a less permeable active acid species, effectively “locking” the drug at the target site. In a recent Phase 1b/2a trial for Parkinson’s disease, NT-0796 not only reduced inflammatory cytokines in Cerebrospinal Fluid (CSF) but also significantly lowered levels of Neurofilament Light Chain (NfL), providing the first clinical proof-of-concept that oral NLRP3 inhibition can attenuate neuronal damage in humans (114).

In parallel, other industry candidates are leveraging structural biology to enhance selectivity and potency. VENT-02, discovered using a proprietary structural platform, has demonstrated a “best-in-class” potency profile with full target engagement and excellent brain penetrance, currently progressing through Phase 2 trials. Additionally, Roche’s Selnoflast is utilizing translocator protein (TSPO) positron emission tomography (PET) imaging in clinical trials to directly visualize its effect on microglial activation in the living brain (110). These advancements signal a shift from preclinical validation to clinical translation, where potent, brain-penetrant NLRP3 inhibitors are poised to modify the disease trajectory by breaking the cycle of neuroinflammation and neurodegeneration.

#### 3.2.3 Targeting the P2X7 receptor to prevent inflammasome activation

The P2X7R acts as a critical sentinel within the microglial neuroimmune response, serving as the primary link between extracellular damage signals and the explosive activation of the NLRP3 inflammasome. Unlike other purinergic receptors that function at physiological ATP levels, P2X7R has a high activation threshold, responding specifically to the millimolar concentrations of eATP found in the “purinergic halo” surrounding necrotic cells and amyloid plaques.1 Upon sustained activation by this pathological stimulus, the receptor undergoes a structural dilation to form a non-selective “macropore,” which facilitates massive potassium (K^+^) efflux and calcium (Ca^2+^) influx (115, 116). This ionic deregulation is the indispensable second signal required for NLRP3 inflammasome assembly, effectively positioning P2X7R as the “upstream trigger” that drives the maturation and release of IL-1β and IL-18 (117).

In the context of AD pathology, P2X7R function shifts from homeostatic maintenance to neurodegenerative toxicity. Histological studies confirm that the receptor is aggressively upregulated in the AD brain, particularly in activated microglia clustering around senile plaques, where expression levels correlate with amyloid load and disease severity (118, 119). While basal P2X7R activity acts as a “scavenger” facilitating cytoskeletal reorganization for phagocytosis, the chronic hyperactivation observed in AD creates a functional paradox. The formation of the transmembrane macropore induces pyroptosis—a highly inflammatory form of cell death—and disrupts the metabolic machinery required for engulfment (117). Consequently, overstimulated microglia become effectively paralyzed; they are unable to clear amyloid debris while simultaneously propagating a feed-forward inflammatory loop through lysosomal destabilization and cytokine release (115, 120).

Therapeutic strategies targeting P2X7R aim to sever this connection between damage sensing and the inflammatory cascade without compromising the receptor’s basal physiological roles. Early interventions using the dye-derivative Brilliant Blue G (BBG) successfully reduced neuronal loss and intracellular Aβ accumulation in mouse models, though its clinical translation was hindered by toxicity and species-specific potency issues (121, 122). The field has since advanced to second-generation, BBB-penetrant antagonists such as the JNJ series (e.g., JNJ-54175446), which have demonstrated robust target engagement and safety in human clinical trials (123). By blocking the receptor’s pore-forming state, these small molecules offer a multifaceted advantage over downstream cytokine inhibitors: they not only halt the release of IL-1β and IL-18 but also impede the exosomal spreading of tau pathology and reduce oxidative stress, potentially functioning as a “circuit breaker” for chronic neuroinflammation (115, 119).

### 3.3 Restoring metabolic homeostasis and microglial renewal

**Figure 5:**
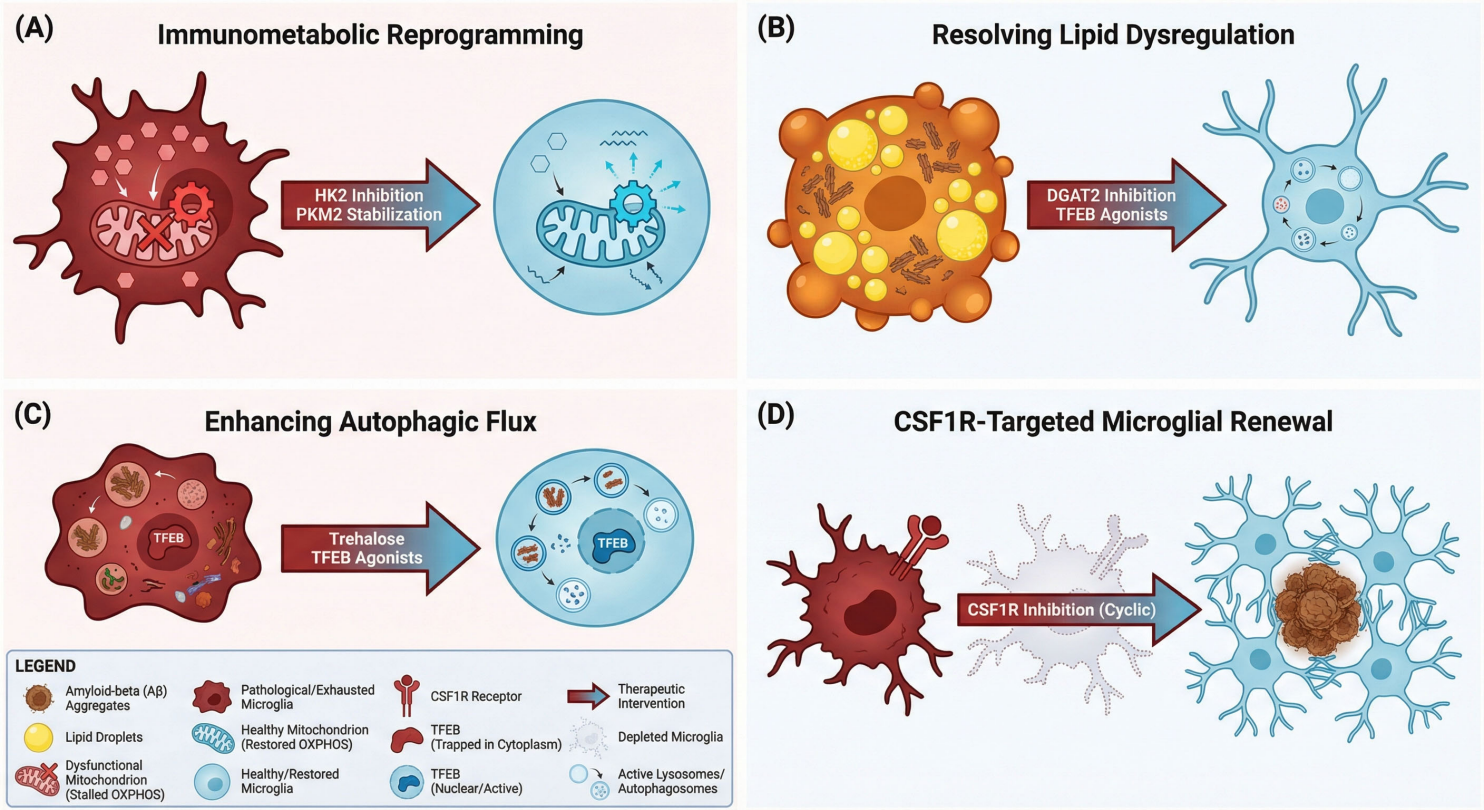
Therapeutic strategies for restoring metabolic homeostasis and microglial renewal in AD. This schematic illustrates four convergent approaches designed to reverse the bioenergetic deficits and functional exhaustion characteristic of AD microglia. Across all panels,metabolically dysregulated or senescent microglia are depicted in dark red/orange,therapeutically restored homeostatic microglia in light blue,and Aβ aggregates in brown. **(A)** immunometabolic reprogramming: The maladaptive “Warburg-like” glycolytic shift in exhausted microglia is reversed by partially inhibiting HK2 or stabilizing PKM2 tetramers. These interventions force a metabolic switch from aerobic glycolysis back to OXPHOS and FAO,thereby restoring energy efficiency and protective phagocytosis. **(B)** resolving lipid dysregulation: LDAMs,which suffer from severe phagocytic blockade due to intracellular lipid congestion,are rescued by inhibiting triglyceride synthesis enzymes (e.g.,DGAT2) or enhancing lipophagy (e.g.,TFEB agonists). This unclogs the cellular machinery,restoring their capacity to clear Aβ. **(C)** enhancing autophagic flux: dysfunctional lysosomes and impaired degradation pathways are rescued via TFEB agonists or the mTOR-independent enhancer trehalose. These agents promote the nuclear translocation of TFEB,reactivating the CLEAR network to facilitate the efficient degradation of engulfed Aβ and suppress inflammasome activation. **(D)** CSF1R-targeted microglial renewal: senescent,pro-inflammatory microglial populations are depleted using cyclic CSF1R inhibition. Upon withdrawal of the inhibitor,the brain is repopulated with healthy,homeostatic microglia capable of effectively compacting Aβ into dense-core plaques and restoring the neuroprotective barrier. Abbreviations: AD,Alzheimer’s disease; Aβ,amyloid-beta; CLEAR,Coordinated Lysosomal Expression and Regulation; CSF1R,Colony-Stimulating Factor 1 Receptor; DGAT2,Diacylglycerol O-acyltransferase 2; FAO,fatty acid oxidation; HK2,Hexokinase 2; LDAM,Lipid Droplet-Accumulating Microglia; mTOR,mammalian target of rapamycin; OXPHOS,oxidative phosphorylation; PKM2,Pyruvate Kinase M2; TFEB,Transcription Factor EB.

Microglial function is inextricably linked to cellular bioenergetics; the high energy demands required for phagocytosis and surveillance cannot be met by cells suffering from metabolic exhaustion or inflexibility. In the AD microenvironment, microglia become metabolically compromised—trapped in a glycolytic state or burdened by lipid droplet accumulation—which renders them functionally inert and pro-inflammatory. Therapeutic strategies are therefore evolving beyond receptor modulation to address these fundamental physiological deficits. Here, we examine approaches aimed at reprogramming immunometabolism, resolving lipid dysregulation in LDAMs, enhancing autophagic flux to clear intracellular debris, and utilizing CSF1R inhibition to deplete senescent populations and stimulate the repopulation of healthy microglia (**Figure 5**).

#### 3.3.1 Immunometabolic reprogramming by targeting glycolysis and HK2

Microglia in AD brain undergo a profound metabolic reprogramming analogous to the Warburg effect in oncology, characterized by a shift from oxidative phosphorylation (OXPHOS) to aerobic glycolysis even under normoxic conditions (124). While initially adaptive for rapid cytoskeletal remodeling, this glycolytic surge becomes maladaptive in the chronic amyloid environment, locking microglia into a pro-inflammatory state driven by the HIF-1α and mTOR signaling pathways (124). Recent epigenetic findings highlight a vicious cycle wherein excess lactate generated by this glycolytic flux promotes histone lactylation (H4K12la) at the promoters of glycolytic genes, effectively cementing this metabolic dysfunction and hindering the cells’ ability to clear pathology (125). Consequently, targeting the enzymatic machinery governing this switch offers a novel avenue to reprogram microglia from a neurotoxic phenotype back to a neuroprotective, phagocytic state.

Among the metabolic regulators, Hexokinase 2 (HK2) has emerged as a critical therapeutic checkpoint due to its pivotal role in gating glucose entry and its specific upregulation in plaque-associated microglia (126). Crucially, therapeutic intervention requires a nuanced approach, often described as a “Goldilocks” effect; complete ablation of HK2 can precipitate metabolic collapse and exacerbate inflammasome activation due to the loss of mitochondrial stability (127). Conversely, partial inhibition—mimicked pharmacologically by agents such as Lonidamine—induces a beneficial metabolic flexibility (126). By constraining glycolytic flux without starving the cell, partial HK2 inhibition compels microglia to utilize alternative fuel sources, specifically driving a shift toward fatty acid oxidation (FAO) and lipid metabolism (128). This metabolic flexibility not only sustains cellular energetics but also enhances the clearance of Aβ via the upregulation of lipid sensors like TREM2, while simultaneously dampening cytokine secretion (126).

Beyond the initial glycolytic entry point, downstream enzymes such as Pyruvate Kinase M2 (PKM2) also present viable targets for modulating immunometabolism. In AD, PKM2 often exists as a dimer that translocates to the nucleus to drive inflammatory gene expression; stabilizing PKM2 into its tetrameric form with small molecules like TEPP-46 can sequester the enzyme in the cytoplasm, thereby restoring metabolic efficiency via the TCA cycle and blocking nuclear inflammatory signaling (129). Collectively, these strategies underscore a paradigm shift in AD therapy: rather than broadly suppressing the immune system, the objective is to metabolically reprogram microglia to correct the bioenergetic deficits that underlie their functional failure. However, translational efforts must account for biological variables, such as the observed sex dimorphism in metabolic susceptibility, where female microglia exhibit a more pronounced glycolytic shift and may require stratified dosing regimens (130).

#### 3.3.2 Resolving lipid dysregulation in lipid droplet-accumulating microglia

Recent single-cell transcriptomic analyses have identified LDAMs as a distinct, maladaptive phenotype that differs fundamentally from the neuroprotective DAM signature. Characterized by the aberrant sequestration of neutral lipids—primarily triacylglycerols—into cytosolic organelles, this state represents a critical metabolic bottleneck linking genetic risk factors, particularly *APOE4*, to immune failure (39). The biogenesis of LDAMs is driven by a specific biochemical cascade: upon encountering fibrillar Aβ, microglia upregulate *ACSL1*, an enzyme that diverts fatty acids from mitochondrial oxidation toward esterification (131). This process is consolidated by the enzyme Diacylglycerol O-acyltransferase 2 (DGAT2), which catalyzes the formation of triacylglycerols, and PLIN2, which stabilizes the resulting droplets (132). In *APOE4* carriers, this metabolic deviation is exacerbated, creating a “genotype-specific” failure where lipids accumulate rather than being transported or metabolized, effectively locking the cell into a dysfunctional state (131).

The functional consequences of this lipid burden are catastrophic for the local neuro-environment, manifesting as a “double hit” of lost protective capacity and gained neurotoxicity. First, LDAMs exhibit severe phagocytic blockade; the physical crowding of the cytoplasm and the metabolic diversion of energy substrates result in cellular “indigestion,” rendering the microglia unable to clear Aβ aggregates or myelin debris (132, 133). Second, these cells transition into a pro-inflammatory senescence-associated state, releasing high levels of cytokines and Reactive Oxygen Species (ROS) due to mitochondrial defects (39, 42). Crucially, LDAMs actively propagate neurodegeneration through dysregulated lipid crosstalk; evidence suggests that *APOE4* LDAMs secrete lipotoxic factors that induce tau hyperphosphorylation in neurons, thereby acting as a vector that translates amyloid pathology into tau-mediated toxicity (131, 134).

Therapeutic strategies in this domain focus on “unclogging” the microglial machinery to restore phagocytic function. The most advanced approach involves the direct inhibition of triglyceride synthesis; small molecule inhibitors of DGAT2 have been shown to clear microglial lipid droplets and significantly reduce amyloid plaque load in preclinical models (132). Precision medicine approaches are also exploring the inhibition of ACSL1 (e.g., via Triacsin C) to prevent the initial metabolic diversion in *APOE4* carriers, potentially shielding neurons from lipotoxicity without broadly suppressing lipid metabolism (131). Furthermore, strategies that enhance catabolism, such as Transcription Factor EB (TFEB) agonists that promote “lipophagy” (the autophagic degradation of lipid droplets), offer a complementary route to resolve the intracellular lipid backlog and re-engage the cell’s degradative pathways (135).

#### 3.3.3 Enhancing autophagic flux via TFEB and trehalose

The functional integrity of microglia in AD is inextricably linked to the Autophagy-Lysosomal Pathway (ALP), which is required not only for the degradation of phagocytosed Aβ but also for maintaining immunometabolic homeostasis (136, 137). In the AD microenvironment, this system frequently collapses due to the dysregulation of TFEB, the master regulator of the Coordinated Lysosomal Expression and Regulation (CLEAR) network (138). Under chronic neuroinflammation, hyperactive signaling via mTORC1 and GSK3β results in the inhibitory phosphorylation of TFEB, sequestering it in the cytoplasm and preventing the transcription of genes essential for lysosomal acidification and biogenesis (139, 140). Therapeutic strategies employing TFEB agonists, such as celastrol or the curcumin analog C1, aim to bypass this blockade. These agents have shown efficacy in restoring lysosomal pH, upregulating the degradative machinery, and re-establishing the phagocytic clearance of protein aggregates in preclinical models (138, 141).

Parallel to transcriptional modulation, the disaccharide trehalose represents a distinct class of autophagy enhancers that operates via an mTOR-independent mechanism. Trehalose functions as a competitive inhibitor of the SLC2A8 (GLUT8) transporter, inducing a state of cellular pseudo-starvation that activates AMPK and subsequently ULK1 to initiate autophagosome formation (142). This mechanism is particularly critical for resolving the newly identified LDAM phenotype, a dysfunctional state characterized by *ACSL1* expression and lipid metabolic failure, often exacerbated in *APOE4* carriers (131). By stimulating lipophagy, trehalose facilitates the catabolism of accumulated lipid droplets and dysfunctional mitochondria (143). This restoration of autophagic flux exerts a dual protective effect: it clears intracellular cargo and simultaneously suppresses the activation of the NLRP3 inflammasome by removing endogenous triggers such as mitochondrial ROS and leaking cathepsins (99, 144, 145).

Despite robust preclinical evidence, the clinical translation of autophagy enhancers has historically been hindered by pharmacokinetic barriers, most notably the rapid degradation of oral trehalose by the intestinal enzyme trehalase. To address this, current clinical efforts focus on advanced delivery methods, such as SLS-005 (intravenous trehalose), which is currently being evaluated in a Phase 2/3 trial (NCT05332678) (146). This investigational approach utilizes high-dose systemic administration to saturate enzymatic degradation and achieve therapeutic CNS concentrations. If successful, this trial would provide biological proof-of-concept that restoring microglial autophagy can reduce Aβ and tau pathologies while resolving the chronic neuroinflammatory milieu (147).

#### 3.3.4 Microglial depletion and repopulation strategies targeting CSF1R

The CSF1R signaling axis serves as the primary survival switch for microglia, regulating their proliferation and viability through the binding of ligands CSF1 and IL-34 (148). Pharmacological blockade of this receptor, utilizing small-molecule inhibitors such as PLX5622 or the clinically relevant JNJ-40346527, triggers rapid, non-inflammatory apoptosis of the microglial compartment, effectively “scrubbing” the brain of dysregulated or senescent immune populations (149, 150). The therapeutic rationale rests on the concept of “microglial resetting”: upon withdrawal of the inhibitor, the CNS is rapidly repopulated by the clonal expansion of residual surviving cells (151). Crucially, these repopulating microglia often exhibit a renewed, homeostatic transcriptomic signature that resembles a younger phenotype, effectively reversing age-associated inflammatory markers and restoring synaptic plasticity (152, 153). This strategy offers a unique opportunity to eliminate the chronic, maladaptive neuroinflammation characteristic of AD without permanently compromising the brain’s innate immune defense.

However, the application of CSF1R inhibition in AD models reveals a complex, stage-dependent dichotomy, particularly regarding amyloid versus tau pathology. While depletion consistently arrests the trans-synaptic propagation of tau and rescues neurodegeneration in tauopathy models, its impact on amyloidosis is fraught with trade-offs (154, 155). Prophylactic depletion can prevent the initial seeding of plaques, but removing microglia in established amyloid pathology disrupts the protective “plaque compaction” barrier (17). This loss of containment leads to diffuse plaque morphology, exacerbated neuritic dystrophy, and a dangerous redistribution of amyloid to the vasculature, potentially precipitating Cerebral Amyloid Angiopathy (CAA) (46, 47). Consequently, the field is pivoting away from chronic ablation toward “cyclic depletion-repopulation” strategies. This pulsed approach aims to periodically clear senescent and tau-spreading microglia while allowing the repopulated cells to return and maintain essential barrier functions, thereby balancing neuroprotective renewal with plaque containment (149, 156).

### 3.4 Advanced delivery and combination therapies

The identification of promising molecular targets addresses only half of the therapeutic challenge; the effective translation of these insights into clinical reality is hindered by the formidable BBB and the multifactorial nature of AD pathology. Traditional systemic administration often fails to achieve therapeutic concentrations in the CNS, and targeting a single pathway may prove insufficient given the complexity of the disease. To overcome these translational hurdles, this final section evaluates cutting-edge engineering solutions, including nanoparticle-mediated delivery systems designed to cross the BBB and target microglia specifically, as well as emerging combinatorial strategies that synergize immunotherapy with metabolic or inflammatory modulation to optimize clinical outcomes.

#### 3.4.1 Nanoparticle-mediated systems for precision microglial targeting

The clinical translation of microglial immunomodulators has historically been impeded by the “double barrier” challenge: the highly selective BBB that excludes most macromolecular therapeutics, and the difficulty of selectively targeting the microglial population (10–15% of brain cells) without off-target effects on neurons or astrocytes (157, 158). To overcome these obstacles, advanced nanoparticle systems have been engineered to exploit physiological transport mechanisms, primarily receptor-mediated transcytosis (RMT). While traditional strategies targeted the Transferrin Receptor (TfR), recent innovations utilize ligands such as the T7 peptide, which binds to a distinct pocket on TfR to avoid competition with endogenous transferrin, thereby significantly enhancing BBB penetration (159). Similarly, targeting the LRP1 receptor with high-affinity peptides like Angiopep-2 has enabled liposomal carriers to achieve superior transcytosis rates (160). These physicochemical modifications allow nanocarriers to navigate the neurovascular unit, serving as a prerequisite for delivering therapeutic cargoes to the brain parenchyma.

Upon traversing the BBB, the next critical objective is achieving precise “homing” to activated microglia, often by hijacking their intrinsic phagocytic and scavenger receptors (161). Strategies utilizing ligand-based targeting, such as mannose-coated lipid nanoparticles (MLNPs), have demonstrated remarkable specificity by engaging the CD206 receptor, achieving high co-localization with microglia while sparing neurons (162). Furthermore, biomimetic approaches are being developed to disguise nanoparticles with “eat-me” signals; for instance, coating particles with Gas6-overexpressing membranes allows them to engage TAM receptors (Tyro3, Axl, MerTK), effectively mimicking apoptotic debris to trigger specific engulfment (163). To resolve the “target conflict” between BBB entry and microglial uptake, sequential “sheddable” systems have been designed. These sophisticated constructs, such as the Res@TcMNP/ASO system, feature a pH-sensitive outer shell that facilitates BBB crossing via TfR but sheds within the endosome to reveal a secondary ligand (e.g., MG1) optimized for microglial binding, ensuring multi-stage delivery efficiency (164).

**Figure 6:**
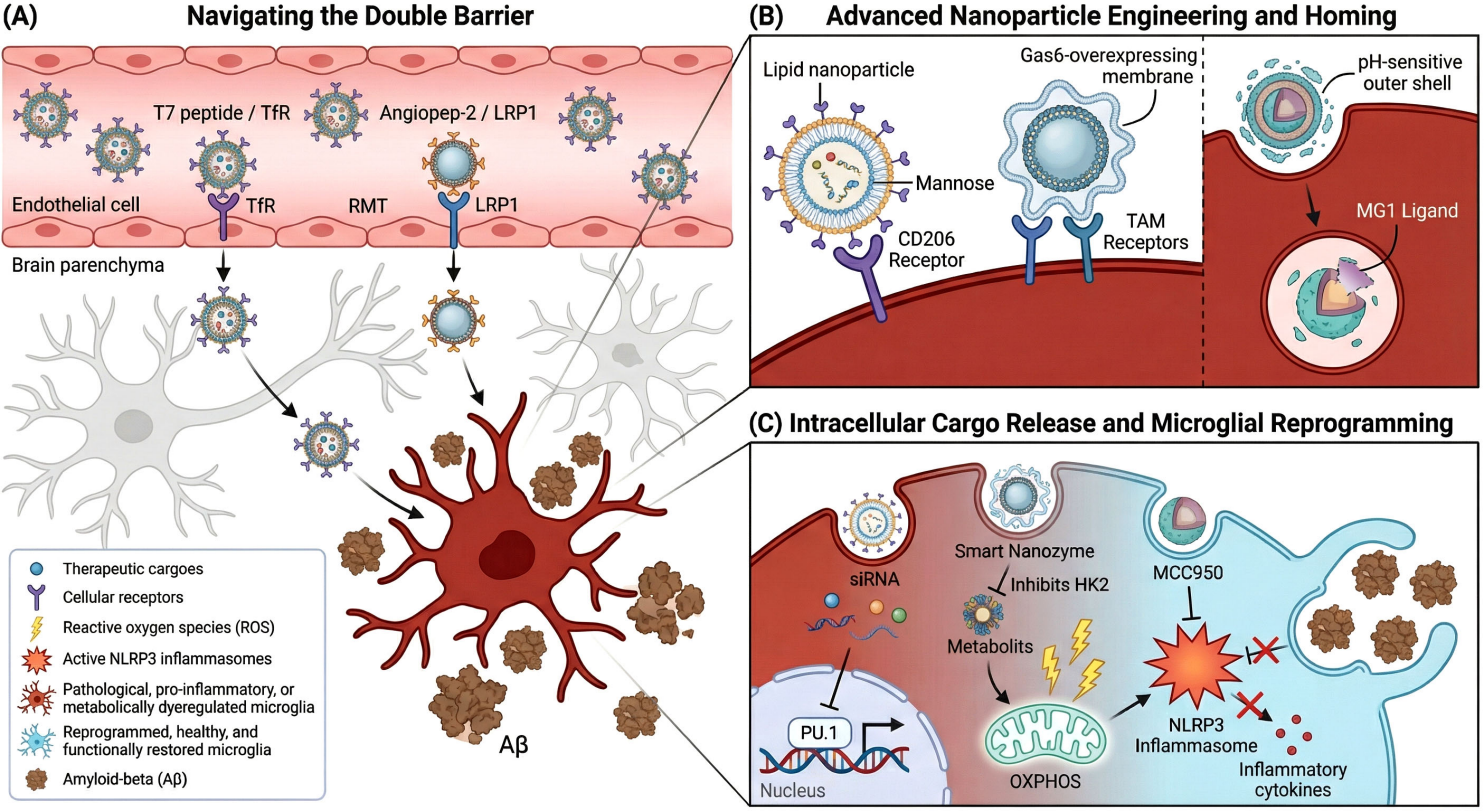
Advanced nanoparticle-mediated delivery systems for precision targeting and phenotypic reprogramming of microglia in Alzheimer’s disease. **(A)** Navigating the double barrier. Engineered nanocarriers overcome the primary blood-brain barrier (BBB) utilizing receptor-mediated transcytosis (RMT) via specific surface modifications,such as the T7 peptide targeting the transferrin receptor (TfR) and Angiopep-2 targeting LRP1. Upon entering the brain parenchyma,these functionalized nanoparticles bypass neurons and astrocytes (gray) to specifically home in on disease-associated,pro-inflammatory microglia (dark red) clustered around amyloid-beta (Aβ) plaques (brown). **(B)** advanced nanoparticle engineering and homing. Diverse functionalization strategies ensure precise microglial engagement: lipid nanoparticles utilize mannose to bind CD206 receptors; biomimetic nanoparticles coated with Gas6-overexpressing membranes exploit TAM receptors by mimicking apoptotic “eat-me” signals; and sophisticated “sheddable” systems employ a pH-sensitive outer shell that dissolves within acidic endosomes to reveal a secondary ligand (e.g.,MG1) for targeted intracellular delivery. **(C)** Intracellular cargo release and microglial reprogramming. The release of customized therapeutic cargoes drives a profound phenotypic shift from a neurotoxic,pro-inflammatory state (left,dark red) to a homeostatic,reparative state (right,light blue). Targeted siRNAs silence the master inflammatory transcription factor PU.1; “smart nanozymes” inhibit glycolysis (via HK2 blockade),scavenge reactive oxygen species (ROS),and restore oxidative phosphorylation (OXPHOS); and inhibitors like MCC950 neutralize the NLRP3 inflammasome to halt inflammatory cytokine release. This integrated genetic,metabolic,and immunomodulatory reprogramming allows microglia to efficiently phagocytose and clear Aβ aggregates without triggering downstream neurotoxicity.

The versatility of these nanoplatforms has facilitated a shift from simple drug delivery to complex cellular reprogramming, targeting the genetic and metabolic drivers of neuroinflammation. Lipid nanoparticles (LNPs) optimized for microglial transfection have successfully delivered siRNAs to silence the master regulator PU.1, dampening inflammatory transcriptional signatures, and delivered antagomirs against miR-17 to restore autophagic flux (162, 165). In the realm of immunometabolism, “smart” nanozymes like the LND@HMPB-T7 system not only inhibit HK2 to switch microglial metabolism from glycolysis back to OXPHOS but also scavenge the resulting reactive ROS, effectively uncoupling phagocytic clearance from cytokine production (159). Additionally, encapsulating inflammasome inhibitors like MCC950 allows for the concentrated blockade of NLRP3 signaling, preventing pyroptosis and enhancing amyloid clearance without systemic immunosuppression (166). Collectively, these nanoparticle-mediated strategies represent a paradigm shift towards precision nanomedicine, offering the potential to arrest the neuroinflammatory cascade by directly remodeling microglial function (**Figure 6**).

#### 3.4.2 Synergizing amyloid clearance with microglial immunomodulation

The recent clinical validation of anti-amyloid mAbs, such as lecanemab and donanemab, marks a paradigm shift in AD therapeutics; however, the modest clinical efficacy observed in Phase 3 trials underscores the limitations of targeting proteinopathy in isolation (4, 5). While these agents effectively reduce plaque burden, clinical data suggest that amyloid clearance alone is insufficient to fully arrest the neurodegenerative cascade once it has been initiated (167). A significant challenge remains the “therapeutic gap,” where disease progression persists despite amyloid removal, likely due to the multifaceted nature of AD pathophysiology involving downstream tau propagation and chronic neuroinflammation (167, 168). Furthermore, the administration of passive immunotherapies is frequently complicated by ARIA, a dose-limiting adverse event driven by perivascular inflammation and BBB compromise (169). Consequently, the field is shifting toward combinatorial regimens designed to synergize mechanical plaque clearance with targeted immunomodulation, aiming to enhance efficacy while mitigating vascular toxicity.

To maximize the therapeutic potential of anti-amyloid antibodies, combinatorial strategies focus on optimizing the functional state of microglia, which effectuate plaque phagocytosis. The efficacy of immunotherapy relies on the responsiveness of the host’s endogenous myeloid cells; however, chronic AD pathology is often associated with dysregulated signaling pathways that may impede efficient clearance (124, 168). Strategies targeting the TREM2-CD33 axis propose a dual mechanism: utilizing TREM2 agonists to sustain essential intracellular signaling (PI3K-mTOR) for cell survival and phagocytosis, while concurrently antagonizing the inhibitory CD33 receptor to lower the activation threshold (70, 78). Complementing this signaling modulation, metabolic reprogramming represents another critical avenue. Glucagon-like peptide-1 (GLP-1) receptor agonists have been shown to modulate microglial immunometabolism, potentially shifting cells from a pro-inflammatory glycolytic profile toward OXPHOS (170). This metabolic restoration could provide the bioenergetic support necessary for microglia to sustain the high energy demands of antibody-mediated plaque clearance and subsequent tissue repair.

**Figure 7:**
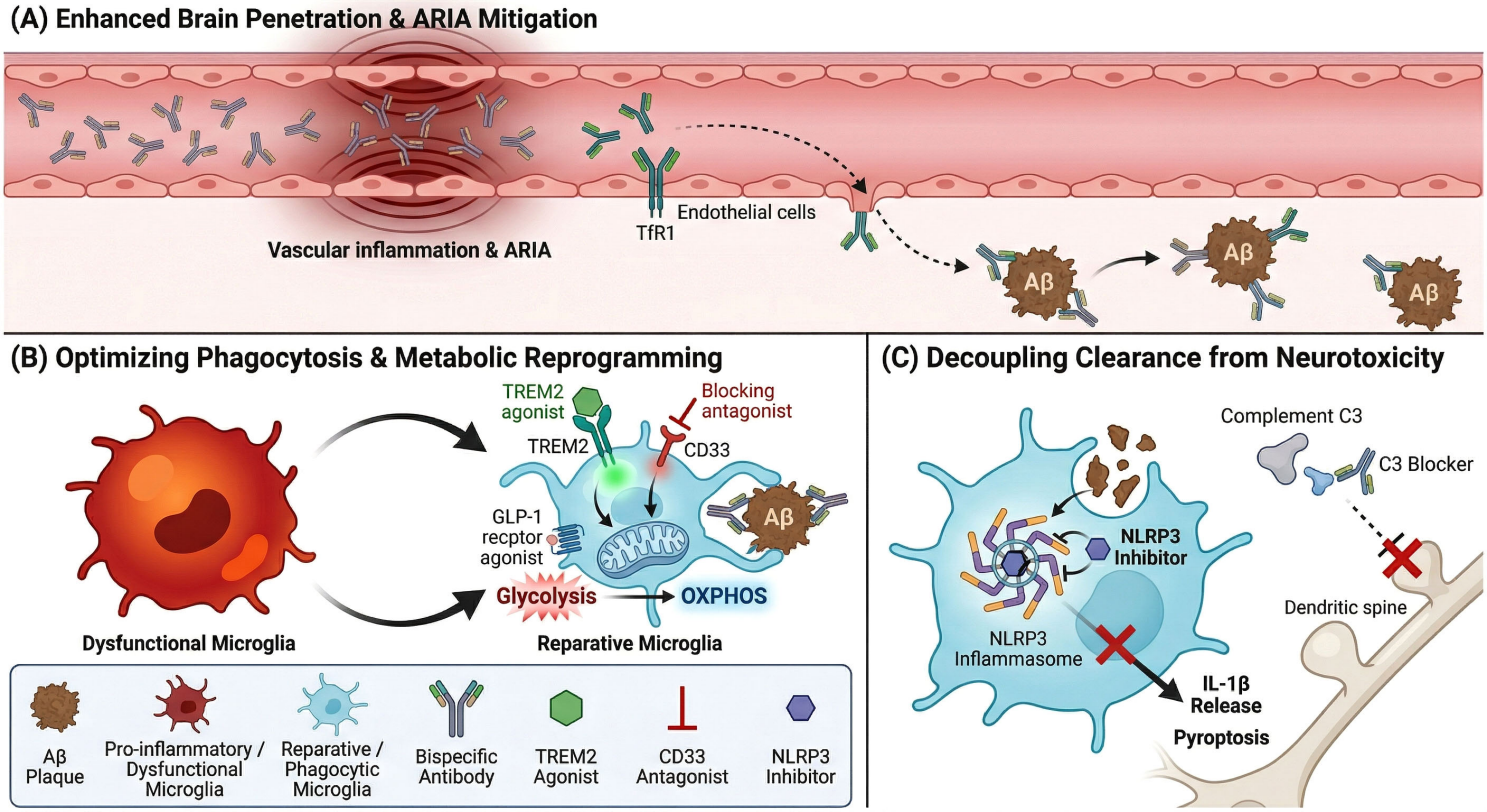
Combinatorial therapeutic strategies synergizing amyloid clearance and microglial immunomodulation. This schematic illustrates advanced therapeutic frameworks designed to overcome the translational limitations and adverse effects of isolated anti-amyloid monotherapies by integrating specific immunometabolic modulators. **(A)** Enhanced brain penetration & ARIA mitigation: Traditional anti-amyloid monoclonal antibodies (mAbs) frequently accumulate in the cerebral vasculature,triggering perivascular inflammation and dose-limiting Amyloid-related imaging abnormalities (ARIA). To circumvent this,engineered bispecific antibodies are utilized to target the Transferrin receptor 1 (TfR1) on endothelial cells. This engagement facilitates efficient receptor-mediated transcytosis (RMT) across the blood-brain barrier (BBB),enhancing drug delivery to the brain parenchyma to tag Aβ plaques while minimizing vascular toxicity. **(B)** Optimizing phagocytosis & metabolic reprogramming: Chronic disease pathology drives microglia into a metabolically exhausted,dysfunctional state (depicted in red/orange). Combinatorial strategies reprogram these cells into a reparative,highly phagocytic phenotype (light blue). This involves lowering the phagocytic threshold through concurrent TREM2 agonism and CD33 antagonism. Furthermore,GLP-1 receptor agonists are employed to correct metabolic dysregulation,shifting the cellular bioenergetics from pro-inflammatory glycolysis back to oxidative phosphorylation (OXPHOS),thereby sustaining the high energy demands of antibody-mediated plaque clearance. **(C)** Decoupling clearance from neurotoxicity: To prevent the innate immune response from damaging healthy tissue during active plaque removal,adjunctive therapies block downstream inflammatory cascades. The application of small-molecule NLRP3 inhibitors prevents inflammasome assembly during Aβ internalization,safely uncoupling phagocytosis from pyroptosis and IL-1β release. Simultaneously,neutralizing the Complement C3 pathway effectively prevents aberrant microglial synaptic pruning,ensuring the preservation of dendritic spines and overall neuronal integrity.

Beyond enhancing clearance efficiency, combinatorial approaches are essential for decoupling phagocytosis from neurotoxic inflammatory responses. The internalization of amyloid aggregates can trigger the assembly of the NLRP3 inflammasome, leading to the release of pro-inflammatory cytokines (IL-1β) and pyroptosis, which may exacerbate neuronal damage and BBB dysfunction (98). The co-administration of small molecule NLRP3 inhibitors aims to suppress this deleterious cascade without impairing the phagocytic uptake of plaques (171, 172). Additionally, inhibiting the complement cascade (specifically C3) may serve as a protective strategy to prevent aberrant synaptic pruning that can occur during the active phase of plaque removal (173). Finally, engineering advancements such as bispecific antibodies targeting the Transferrin Receptor 1 (TfR1) facilitate RMT (174). By enhancing brain parenchymal delivery, these novel vehicles reduce the requirement for high systemic dosing, thereby minimizing the vascular accumulation associated with ARIA and improving the overall safety profile of combinatorial interventions (**Figure 7**) (175).

### 3.5 Microglial senescence phenotypes and targeted senotherapeutic strategies

As the brain ages and neurodegenerative pathologies progress, a subset of microglia undergoes irreversible cell cycle arrest, transitioning into a senescent state characterized by a profound loss of homeostatic functions and the adoption of a senescence-associated secretory phenotype (SASP) (176). Morphologically, these cells frequently present a dystrophic phenotype, exhibiting deramification, process shortening, beading, and cytoplasmic fragmentation (177). Beyond structural alterations, senescent microglia suffer from severe metabolic and phagocytic impairments, including dysregulated lipid metabolism that leads to the generation of lipid droplet-accumulating microglia, as well as a marked intracellular accumulation of iron sequestered within ferritin molecules (178, 179). In the context of Alzheimer’s disease, prolonged exposure to both Aβ and pathological tau drives this microglial senescence, trapping the cells in a chronic pro-inflammatory state where they lose their ability to clear protein aggregates and instead secrete cytokines, chemokines, and proteases that exacerbate neurotoxicity and actively drive the propagation of the disease (180).

To combat the accumulation of these dysfunctional immune cells, senolytics—small molecules designed to selectively eliminate senescent cells and induce apoptosis—have emerged as a highly promising therapeutic frontier (180). The combination of dasatinib and quercetin (D+Q) is among the most extensively studied senolytic regimens. In preclinical Alzheimer’s models, D+Q treatment successfully cleared p16^INK4A^-positive senescent microglia, dampened neuroinflammation, and reduced Aβ burden, and is currently being evaluated in clinical trials for older adults (ClinicalTrials.gov ID NCT04685590) (181–183). Similarly, the BCL-2 inhibitor Navitoclax (ABT-263) and the targeted apoptosis-inducer AP20187 have demonstrated the ability to eliminate senescent microglia in tauopathy models, halting tau hyperphosphorylation and preventing cognitive decline (184, 185). Furthermore, naturally occurring flavonoids like Fisetin are undergoing pilot clinical trials to assess their efficacy in clearing the senescent microglial burden (186). Beyond molecular senolytics, researchers are exploring radical microglial replacement strategies utilizing CSF1R inhibitors. As discussed in Section 3.3.4, this approach pharmacologically depletes the exhausted, aged microglial population, allowing the brain to repopulate with a youthful, homeostatic microglial compartment, thereby resetting the immune environment and improving synaptic plasticity (187).

Rather than outright eliminating senescent cells, an alternative intervention framework focuses on senomorphics and metabolic rejuvenation to suppress the SASP and restore microglial functionality. Senomorphics, including the mTOR inhibitor rapamycin and NF-κB inhibitors such as resveratrol and apigenin, function by blocking the signaling pathways that maintain the pro-inflammatory secretory profile of senescent microglia, thereby protecting neurons from inflammation-induced death (180). Rejuvenation strategies specifically target the metabolic and phagocytic deficits of dystrophic microglia. For instance, microglial phagocytosis can be restored by inhibiting the CD22 receptor or hexokinase 2, while targeting EP2/4 receptors can revitalize microglial energy metabolism by reprogramming glucose and glycogen utilization (188–190). Given the prominent iron overload in dystrophic microglia, iron chelation therapies utilizing agents like deferoxamine, deferiprone, and the versatile chelator M30 are also being investigated to prevent iron-dependent oxidative stress (179). To overcome the inherent challenge of delivering these diverse therapeutics across the blood-brain barrier specifically to microglia, cutting-edge delivery platforms are being developed, including nanomedicine formulations designed for precise endolysosomal escape, as well as recombinant TREM2-activating antibodies delivered via antibody transport vehicle systems to directly stimulate microglial metabolism and clearance functions (186, 191). Ultimately, as clinical validation progresses, personalized combinations of senolytic and senomorphic treatments may be required for optimal therapeutic outcomes. The greatest promise of studying microglial senescence lies not in reversing time, but in reclaiming the physiological equilibrium of these resident immune defenders to sustain brain health and effectively combat neurodegeneration.

### 3.6 The role of biomarkers and the emerging ATI(N) framework

Initially proposed in 2016 as a biomarker-driven scheme and formally integrated into the updated 2018 National Institute on Aging and Alzheimer’s Association (NIA-AA) research framework, the AT(N) classification system classically defined Alzheimer’s disease based on amyloid deposition, tau pathology, and neurodegeneration (192, 193). While this symptom-agnostic scheme has been fundamental in conceptualizing the spatiotemporal biological evolution of AD, it is increasingly recognized as insufficient to capture the full pathophysiological landscape of the disease. Because Alzheimer’s is deeply multifactorial, researchers have advocated for expanding this inherently flexible model into an ATX(N) classification, where “X” represents novel candidate biomarkers reflecting other critical pathogenic mechanisms (194). Building upon this logical transition, the academic community is actively moving toward the more specific ATI(N) framework, where the “I” distinctly designates immunity and neuroinflammation. This paradigm shift is driven by multifaceted evidence identifying microglial-mediated neuroinflammation as a core, independent driver of disease progression rather than a mere secondary response (195). As microglia undergo complex transcriptomic changes, transitioning from homeostatic states to disease-associated profiles, they dictate the inflammatory environment of the brain (49). Thus, formally integrating immunity into the diagnostic criteria is essential for a comprehensive biological understanding of the illness.

Integrating the “I” component into standard clinical evaluation relies on a robust panel of fluid and imaging biomarkers that mechanistically reflect glial activation. For example, sTREM2 in the cerebrospinal fluid has emerged as a reliable dynamic indicator of microglial activity, correlating with plaque burden and reflecting the transition to disease-associated microglial states (196, 197). Alongside sTREM2, elevated levels of inflammatory cytokines and astrocytic markers like glial fibrillary acidic protein (GFAP) and YKL-40 provide a non-invasive means to track how neuroinflammation exacerbates neuronal injury, synaptic loss, and cognitive decline over time (198, 199). In parallel, advanced neuroimaging modalities add an essential layer of spatial precision to this framework. TSPO-PET enables the direct *in vivo* visualization of microglial activation and neuroinflammation across specific cortico-limbic brain regions (56).

Ultimately, the formal adoption of the ATI(N) framework carries profound clinical implications, particularly for the advancement of precision medicine and the design of future clinical trials. Because AD pathology is highly heterogeneous, utilizing this expanded biological profile is vital for accurate patient stratification, reducing the risk of clinical trial failure by identifying populations most likely to respond to targeted therapies (56, 200). Furthermore, biomarkers within the “I” category allow clinicians to assess the functional state of microglia, dynamically classifying patients to determine the optimal temporal window for intervention before irreversible damage occurs. Finally, as novel immune-modulating treatments enter clinical development, such as TREM2-targeted interventions, these specific inflammatory markers are absolutely indispensable for evaluating target engagement and dynamically monitoring real-time therapeutic efficacy.

## 4 Conclusion and future perspectives

The paradigm of AD research has undergone a profound transformation, evolving from a strictly neurocentric view focused on amyloid and tau proteinopathies to a more holistic framework that positions the innate immune system as a central driver of pathogenesis. As detailed in this review, microglia are not merely passive responders to neurodegeneration but are dynamic, plastic orchestrators that can determine the trajectory of the disease. The recognition of their “double-edged” nature—shifting from homeostatic guardians to protective barriers (DAM), and ultimately to chronic executioners of neurotoxicity—provides a critical explanation for the failure of earlier broad-spectrum anti-inflammatory trials. It highlights that the success of immunomodulatory therapies hinges not on suppression alone, but on the precise, stage-dependent modulation of microglial phenotypes.

Moving forward, the clinical translation of microglia-targeted therapies faces several pivotal challenges that define the future research agenda. First, the timing of intervention is paramount. Given the dichotomous role of microglia, therapeutic strategies must be stratified according to disease stage. Early-stage interventions might focus on enhancing TREM2-mediated chemotaxis and phagocytosis to contain nascent pathology, whereas late-stage therapies should prioritize the blockade of the NLRP3 inflammasome to arrest catastrophic neuroinflammation and synaptic loss. As detailed in Section 3.5, operationalizing the ATI(N) framework through the integration of fluid and imaging biomarkers—specifically those reflecting microglial activation states (e.g., sTREM2, TSPO-PET, and novel inflammasome markers)—will be absolutely critical to enabling this kind of true precision medicine.

Second, the complexity of immunometabolism offers a promising but under-explored frontier. Restoring the metabolic flexibility of microglia, particularly by correcting the glycolytic shift and resolving lipid droplet accumulation in *APOE4* carriers, represents a novel strategy to rejuvenate senescent immune functions from within. Future therapeutics will likely move beyond cell surface receptors to target these intracellular metabolic checkpoints (e.g., HK2, mTOR, TFEB) to restore autophagic flux and phagocytic competence. Furthermore, the targeted clearance or rejuvenation of dystrophic, senescent microglia using emerging senolytic and senomorphic agents holds immense promise for fundamentally resetting the aging neuroimmune environment.

Finally, the era of monotherapy is likely yielding to combinatorial approaches. The future standard of care for AD will likely involve synergistic regimens: using anti-Aβ antibodies to directly clear pathological amyloid aggregates, while simultaneously deploying microglial modulators to mitigate adverse inflammatory events (such as ARIA and cytokine release) and facilitate tissue repair. Coupled with advancements in BBB-crossing nanoplatforms that ensure targeted and efficient delivery, these strategies offer renewed hope. By mastering the intricate language of microglial regulation, we stand on the precipice of transforming AD from an inevitable neurodegenerative decline into a manageable chronic condition.
